# Artificial intelligence and computational methods in the Asia-Pacific pharmacovigilance landscape: a systematic review

**DOI:** 10.3389/ijph.2026.1610026

**Published:** 2026-09-11

**Authors:** Gita Kusnadi, Grace Wangge, Arif Perdana

**Affiliations:** Monash University (Indonesia), Bumi Serpong Damai, Indonesia

**Keywords:** AI, artificial intelligence, Asia-pacific, drug safety, pharmacovigilance

## Abstract

**Objective:**

To examine the application of artificial intelligence (AI) in pharmacovigilance across the Asia-Pacific and identify reported methodological implementation challenges.

**Methods:**

MEDLINE, Scopus, and Google Scholar were searched using terms related to artificial intelligence, computational signal detection, pharmacovigilance, and Asia-Pacific countries. Peer-reviewed original studies published in English were included. PRISMA 2020 guideline was followed.

**Results:**

We included 64 studies in 14 countries primarily focused on 1) Adverse Drug Reaction (ADR) identification, 2) ADR prediction and risk factor modelling, 3) Drug safety, monitoring, and evaluation, 4) Predictive modelling, and 5) Data information management. Machine Learning (ML) techniques were the most commonly applied AI methods in pharmacovigilance, followed by natural language processing, deep learning, neural networks, and symbolic and explainable AI. Disproportionality analysis methods were also commonly used across studies. Some challenges reported were relevant to data quality issues, generalizability, clinical workflow integration, implementation technicalities, and cultural barriers.

**Conclusion:**

To overcome the challenges of AI application in the Asia-Pacific, a tiered implementation strategy can be employed through establishing a regional collaboration framework and taking into account disparities in technological maturity across countries.

## Introduction

The Asia-Pacific region has recently experienced rapid growth in its pharmaceutical market. In 2021, the production output of pharmaceuticals and medical equipment in the Asia-Pacific was ranked the highest globally [1]. Due to its large population, efforts to expand healthcare access, and business improvements, it is expected that the region will experience the fastest growth in pharmaceutical and medical equipment production from 2021 to 2030 [1]. As production and consumption expand across the region, so does the volume of medicines in real-world circulation, making robust post-marketing pharmacovigilance an increasingly essential safeguard. Yet pharmacovigilance systems across the Asia-Pacific remain at a nascent stage and consistently fall short of the World Health Organization (WHO) performance standards [2]. This has urged the immediate implementation of a robust pharmacovigilance system in the region.

Since the 55th World Health Assembly took place in 2002, more attention has been paid to patient safety in the healthcare system [3]. Nonetheless, as important as prioritizing patient safety through a robust pharmacovigilance system, implementing robust pharmacovigilance is not without any challenges in the Asia-Pacific. While some countries like Japan and Korea have comprehensively established pharmacovigilance ecosystems, some others are still in the stage of developing the system owing to some challenges such as healthcare infrastructure, regulatory, cultural, and linguistic barriers, and economic developments [2]. This diversity has led to remarkable disparities in pharmacovigilance practices, structures, and outcomes across the countries in the region.

In addition to the general challenges in pharmacovigilance -such as underreporting of adverse drug reactions (ADRs), limited numbers of trained healthcare professionals, and inadequate financial resources- [4–6], traditional medicine practices in some countries in the Asia-Pacific add to the complexity of pharmacovigilance implementation in the region [7–10]. Renowned for its traditional medicines, such as Traditional Chinese Medicine (TCM) and Kampo Medicine, Asia-Pacific faces unique, yet complicated, challenges in integrating a modern pharmacovigilance framework into these indigenous medicines [11, 12]. The complex formulation of traditional medicines, compounded with poor quality control and distribution channel regulation, poses further challenges in the incorporation of these medicines in the pharmacovigilance system [13].

To tackle those general and context-specific challenges in pharmacovigilance practice, some countries in the Asia-Pacific have started to integrate artificial intelligence (AI) into their pharmacovigilance systems [14, 15]. Recently, AI adoption in pharmacovigilance practices has been reported in some studies to be effective in supporting data automation and extraction from different sources, improving signal detection and reporting in pharmacovigilance systems [16–18]. Moreover, some studies have highlighted the beneficial outcomes of AI application in pharmacovigilance, including improvements in the accuracy and speed of ADR signal detection, as well as support for real-time data analysis [16, 18, 19].

Despite increasing interest in AI-enabled pharmacovigilance, the extent and maturity of its application across the Asia-Pacific remain unclear. Existing reviews have largely examined global evidence or focused on particular data sources and predictive tasks, with limited attention to regional differences in language, traditional medicine, reporting infrastructure, regulatory capacity, and health-system readiness. This review therefore aimed to: (1) map the pharmacovigilance tasks addressed by AI-based and conventional computational methods in Asia-Pacific countries; (2) examine the methods, data sources, and reported outcomes of the included studies; (3) identify methodological, validation, and implementation challenges; and (4) synthesise recommendations reported by the included studies.

## Methods

A systematic review of literature was conducted in Medline, Scopus, and Google Scholar (restricted only to the first 100 results for Google Scholar). The database search was conducted from January to March 2025. The selection of the databases was driven by the broad coverage of biomedical, clinical, public health and interdisciplinary literature discussing pharmacovigilance and artificial intelligence. Google Scholar was additionally searched to enhance the retrieval of multidisciplinary, gray, and rapidly emerging literature, which may enhance the identification of relevant evidence in the rapidly evolving field of artificial intelligence in the region [20]. To ensure that the review captured the most recent evidence, a targeted update search of MEDLINE was conducted in July 2026 (during the publication process of this article). The updated search did not identify any new methodological or conceptual developments that would alter the findings or conclusions of this review.

This systematic review was conducted in accordance with Preferred Reporting Items for Systematic Review and Meta-analyses (PRISMA) guidelines (see Supplementary Material 1) [21]. A keyword combination of “artificial intelligence” AND “pharmacovigilance” AND “Asia-Pacific” along with its synonyms was used to assess the use of artificial intelligence in pharmacovigilance in the Asia-Pacific (Full strategy is presented in Supplementary Material 2). To capture the historical development of computational pharmacovigilance, the review included both conventional statistical signal-detection methods and contemporary AI-based methods. Conventional methods included disproportionality analyses such as reporting odds ratios, proportional reporting ratios, and Bayesian Confidence Propagation Neural Networks. These methods were not classified as AI. AI-based methods included machine learning, deep learning, natural language processing, neural-network models, ontology or knowledge-based systems, and techniques used to explain model outputs. The two groups were analysed separately when describing methodological trends but jointly when synthesising cross-cutting challenges such as data quality, validation, interoperability, and implementation. The Asia-Pacific countries covered in this review were defined by referring to the United Nations Economic and Social Commission for Asia and the Pacific (UN ESCAP) [22].

Studies were eligible if they: (1) reported original, peer-reviewed research; (2) were published in English; (3) used data from at least one UN ESCAP member or associate member; and (4) evaluated an AI-based or conventional computational method for a pharmacovigilance activity concerned with detecting, assessing, understanding, preventing, or managing adverse effects or other medicine-related safety problems. Studies were excluded if they were editorials, opinions, or non-systematic reviews (such as narrative reviews and literature reviews).

Started with comprehensive database research, the review process was then continued with the removal of duplicates and the title and abstract screening. A further comprehensive review was conducted based on the defined eligibility criteria. After all, the following data were extracted from the included studies: authors, year of publication, title, settings or data source, research focus, AI application, type of drugs, main conclusion, challenges of AI use in pharmacovigilance, recommendations, and some specific AI use discussions. Owing to substantial heterogeneity in study objectives, pharmacovigilance tasks, data sources, model types, validation procedures, outcome definitions, and performance measures, quantitative pooling was not considered appropriate. The studies were therefore synthesised narratively and organised according to country, pharmacovigilance task, computational method, data source, validation stage, reported challenge, and recommendation. Methodological categories were defined before synthesis and were not mutually exclusive because some studies combined multiple computational approaches. Challenges and recommendations were coded inductively from the included studies. GK and GW conducted the extraction and coding, and disagreements were resolved through discussion and consensus with the full author team.

### Quality assessment

The quality of the included studies was appraised using critical appraisal tools selected based on the study design and intended purpose of the AI model. A descriptive reporting assessment was conducted using seven reporting domains adapted from previous systematic reviews evaluating AI-based clinical and diagnostic studies [23, 24]. The assessment criteria included conflict of interest disclosure, study aim, input feature, ground truth, dataset distribution, performance metric, and description of the AI model. These seven items were scored on a two-point scale: 0 (not reported or unclear) or 1 (reported and adequate). For those studies developing or validating individualized multivariable diagnostic or prognostic prediction models (n = 14), a supplementary appraisal was conducted using the Prediction Model Risk of Bias Assessment Tool (PROBAST). One randomised study [25], meanwhile, was appraised using Joanna Briggs Institute (JBI) critical appraisal tool [26].

## Results

### Study selection process

**FIGURE 1:**
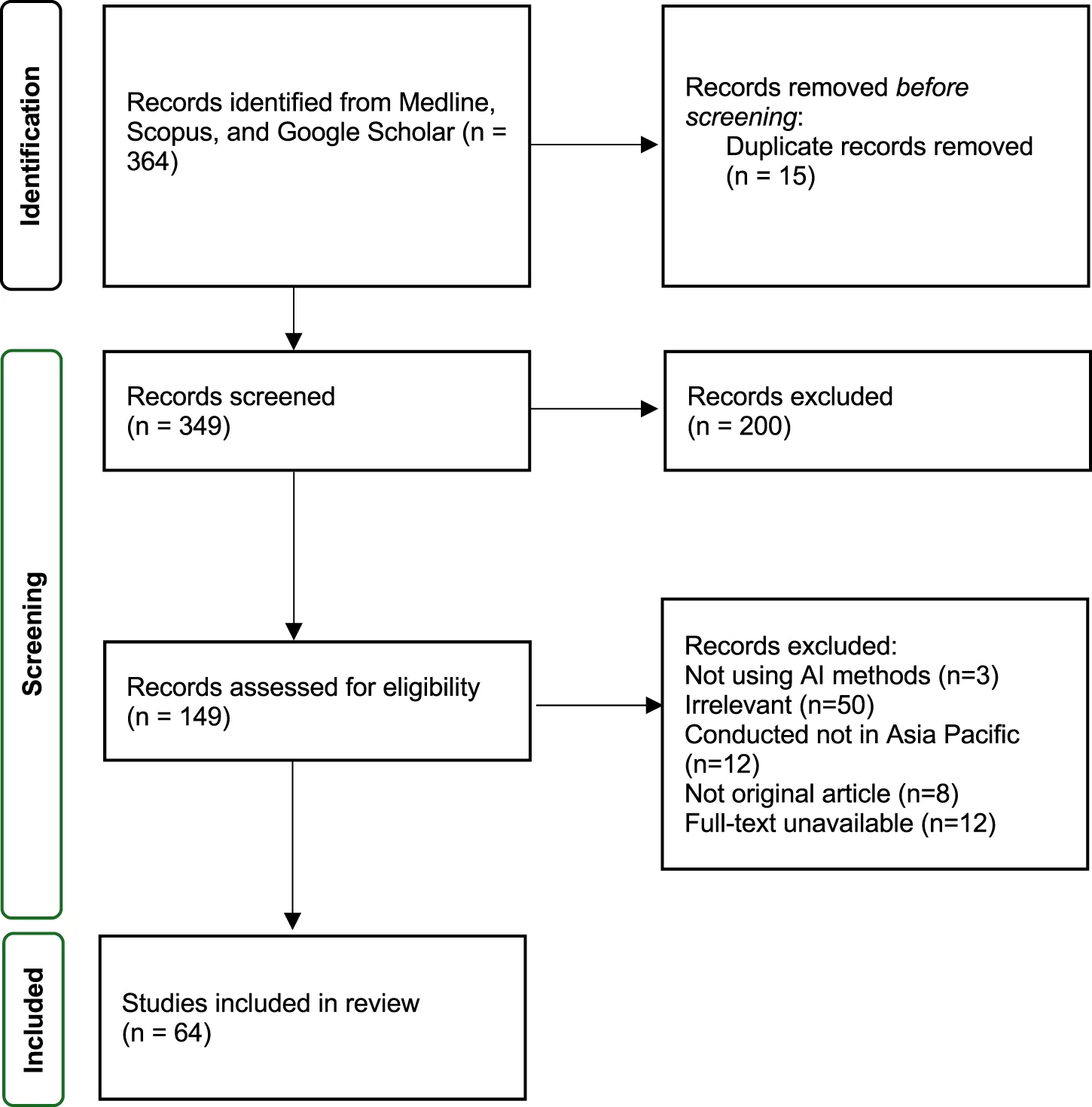
Flow diagram of the study selection process (Asia-Pacific Systematic Review, 2026).

We identified 364 articles through the initial comprehensive database search (Figure 1). Upon removing 15 duplicative articles, 349 articles then went through the title and abstract screening process, where 200 of them were excluded and 149 were thoroughly reviewed against the inclusion criteria. During the process, 85 studies were excluded owing to several different reasons, including: the absence of an AI application in their methodology (n = 3), a lack of relevance (n = 50), being conducted in countries outside the Asia-Pacific region (n = 12), not being primary research (n = 8), or unavailable in full-text (n = 12). Finally, 64 eligible studies were analysed narratively in this review.^25,27–66,67(p019),68–89^

### Quality appraisal

**FIGURE 2:**
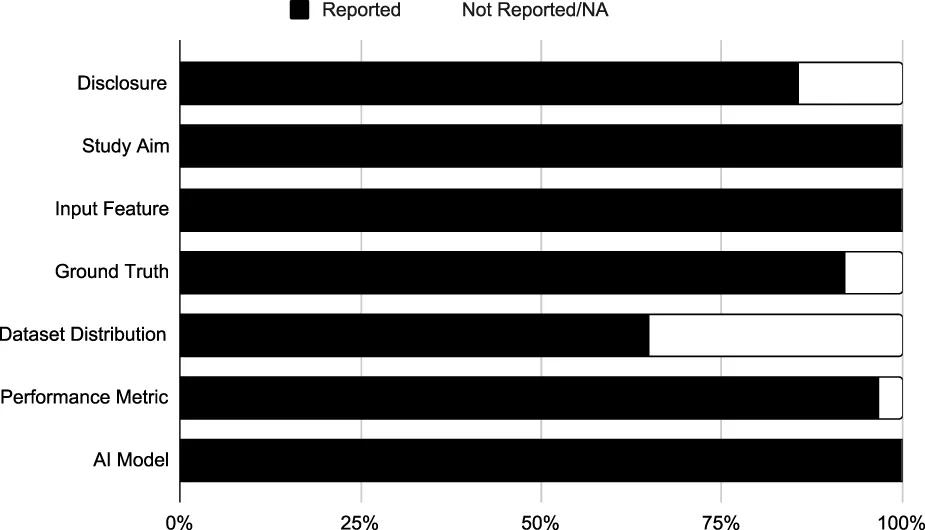
Study quality critical appraisal results (Asia-Pacific Systematic Review, 2026).

Figure 2 demonstrates the results of the quality appraisal for non-randomised studies. Among all assessment domains, the study aim, input features and AI model were clearly reported across all studies. Additionally, 86% of the studies disclosed conflicts of interest involved in their research. Regarding the ground truth, the assessment domain was adequately addressed by 83% of the studies. Lastly, the assessments of dataset distribution and performance metrics were adequately addressed by 65% and 97% of the studies, respectively. This lack of reporting on data distribution, however, should be interpreted cautiously due to the inapplicability of the assessment question in a few studies. While the tool was technically developed to assess studies focusing on the development and training of predictive AI models in health research, several questions were not relevant to real-world implementation AI studies. These included studies focusing on AI operational implementation, signal detection applicability, or the comparison of different data mining methodologies in pharmacovigilance [27–45]. he critical appraisal of the RCT indicated that the study was of a high quality since it met all the criteria in the assessment tool [25].

As for the PROBAST assessment, the results showed that all the 14 predictive modelling studies have a low risk of bias across the participants, predictors, and outcomes domains [29, 46–58]. Nevertheless, the results in the analysis domain indicated greatest methodological limitations, where 79% of the assessed studies (n = 11) were judged to have a high risk of bias primarily due to the unavailability of external validation, inadequate assessment of model optimism, and insufficient reporting of missing-data handling [29, 47, 49, 51–58]. As a result, these 79% studies (n = 11) were judged to have an overall high risk of bias. Regarding the applicability assessment, all studies (n = 14) were considered to have low concerns of applicability [29, 46–58]. The details of the quality appraisal are presented in the Supplementary Material 3.

### Study characteristics

**FIGURE 3:**
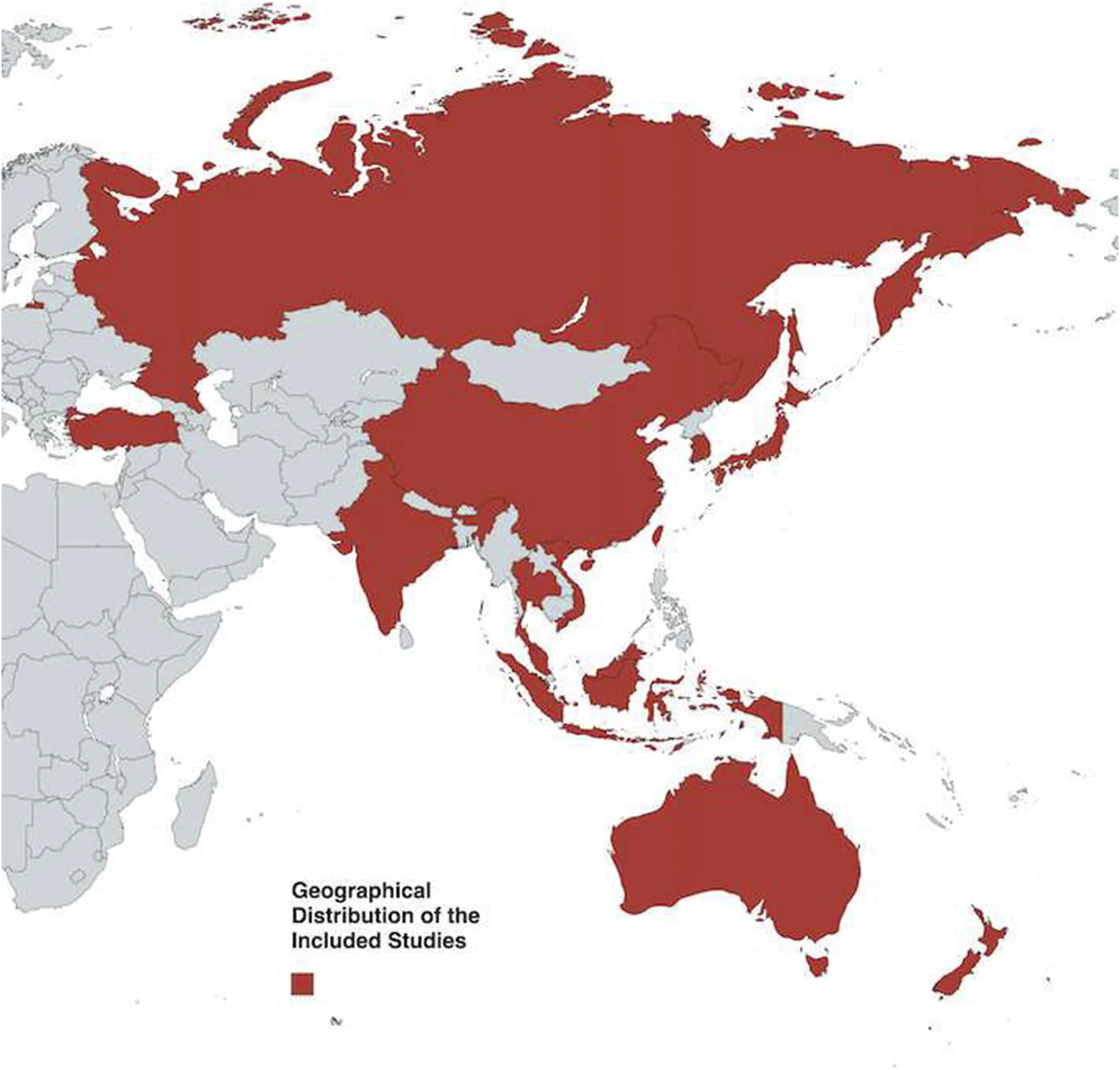
Geographical distribution of studies (Asia-Pacific Systematic Review, 2026).

This systematic review summarised the findings of the studies conducted in 14 different countries in the Asia-Pacific, including China, Japan, Australia, South Korea, Taiwan, Singapore, Malaysia, India, Indonesia, Vietnam, New Zealand, Turkey, Thailand, and Russia (Figure 3). Among these countries, Japan (n = 17) and China (n = 15) are the leading countries in the Asia-Pacific in terms of the number of publications about AI applications in pharmacovigilance, followed by South Korea (n = 6), Taiwan (n = 5), Australia (N = 4), and other remaining countries. A summary of the study characteristics can be found in Supplementary Material 4.

**FIGURE 4:**
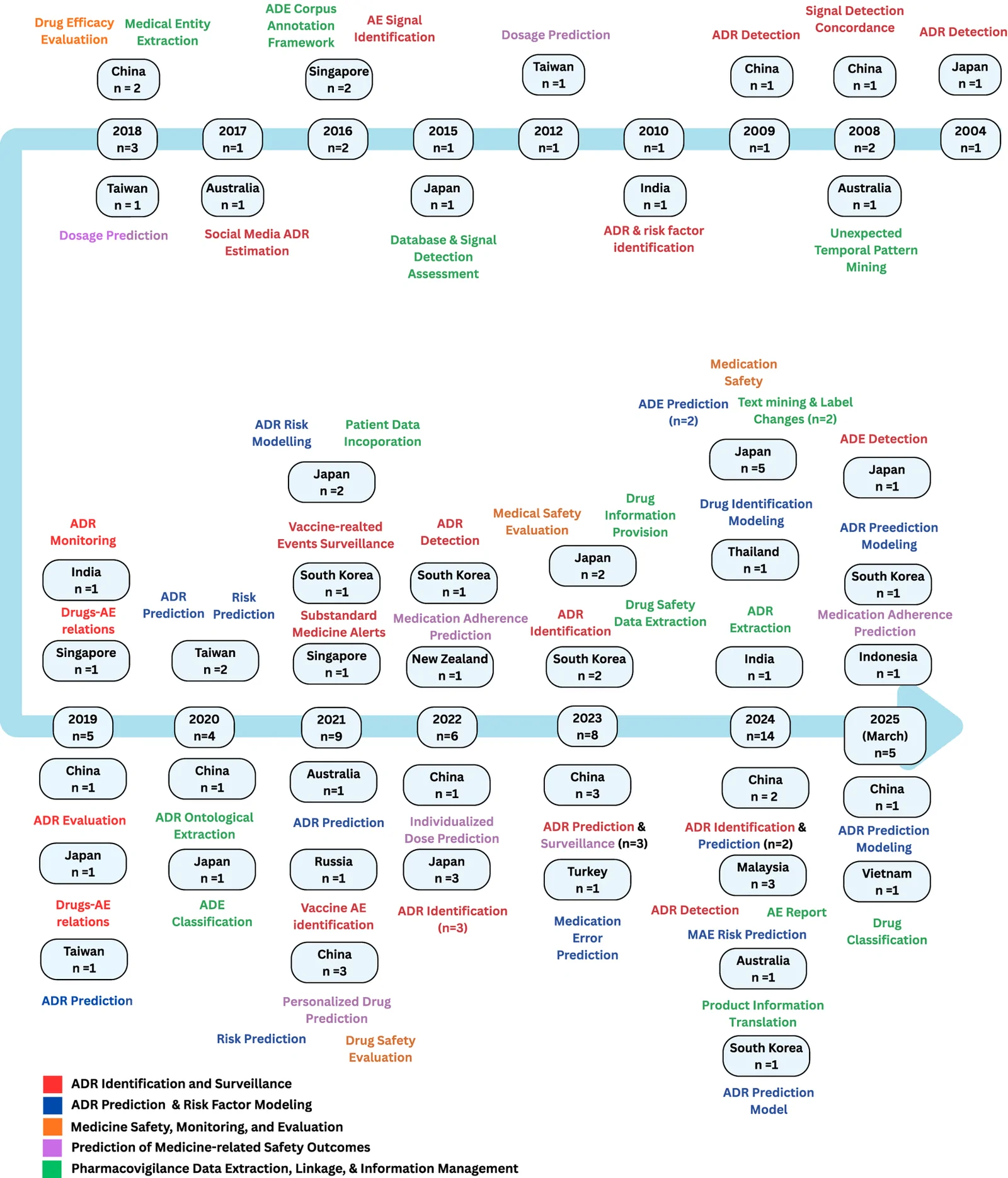
Timeline and focus of AI-Pharmacovigilance Studies in Asia-Pacific (Asia-Pacific Systematic Review, 2026).

In terms of study focus, five broad application areas were identified: 1) ADR identification and surveillance, 2) ADR prediction and risk factor modelling, 3) medicine safety, monitoring, and evaluation, 4) prediction of medicine-related safety outcomes, including toxicity and dose-related risk, and 5) pharmacovigilance data extraction, linkage, and information management. As illustrated in Figure 4, ADR detection and surveillance have consistently remained the primary focus of AI applications in pharmacovigilance across the Asia-Pacific region.

### AI application in the Asia-Pacific pharmacovigilance landscape

Table 1 presents the details of AI applications across a wide range of drugs and medications in the Asia-Pacific and the findings statement reported by the original study authors. Machine-learning methods were reported in 25 of the 64 included studies (39%). Because some studies used more than one computational approach, method categories were non-mutually exclusive and percentages do not sum to 100% [29, 45–49, 51–54, 56, 57, 59–67]. Statistical signal detection, such as Reporting Odds Ratio (ROR), Proportional Reporting Ratio (PRR), and Bayesian Confidence Propagation Neural Network (BCPNN), represented early integration of computational methods in pharmacovigilance system across the Asia-Pacific and laid the foundation for the wider adoption of AI-based methods in the region (Figure 4) [27, 30–32, 37–39, 42, 68, 69]. Most studies reported positive outcomes of applying these disproportionality methods, highlighting their simplicity and efficiency in basic ADR signal detection [27, 32, 37, 38, 42, 68]. However, some other studies had neutral views towards the methods owing to several challenges, such as data variations and sufficiency, as well as lower sensitivity compared to other methods [30, 31, 39, 69].

**TABLE 1:** Characteristics of AI-based and computational methods applied in pharmacovigilance (Asia-Pacific Systematic Review, 2026).

No	Methods*	Specific Techniques	No of Studies	Drugs/Diseases Involved
1	Statistical disproportionality methods	ROR, PRR, MHRA/MCA method, BCPNN, GPS, MGPS, RRR, PRRCI, RORCI	10	General drugs, TCMs, paliperidone palmitate, cardiac therapy drugs, antiretroviral therapyetc.
2	Machine learning (ML)	Random forest, SVM, logistic regression, XGBoost, LightGBM, CatBoost, AdaBoost, TPOT, decision tree, LASSO, ridge regression, GLM, elastic net, extra trees regressor, kNN, SVR, MLP, CART, model tree	25	Antipsychotics, tacrolimus, methotrexate, ICIs, anti-TB drugs, warfarin, digoxin, vaccinesetc.
3	Natural language processing (NLP)	NER, IE, NLC, BERT (tohoku, UTH, ELECTRA, PhoBERT), pretrained NER, NLP + ML	15	General drugs, SLE, chemotherapy, kampo, cancer meds, ADE detection, COVID-19 vaccine, acetaminophen/ibuprofen
4	Deep learning (DL)	Deep neural network (DNN), BiLSTM, BiLSTM-CRF, DCNN, YOLOv8-nano, IMV-LSTM	7	TCM + Parkinson’s, SCARs, DILI, general drugs
5	Neural networks (general)	ANN, GAN, Word2Vec, simple RNN, LSTM, MLP, BiLSTM	9	Parkinson’s, COVID-19, hepatotoxicity, lipid therapy, TCMs
6	Explainable AI (XAI)	LIME, SHAP	2	Vaccines, cardiovascular/musculoskeletal drugs
7	Symbolic AI/KRR/Ontology-based	Ontology-based side-effect prediction (OSPF), KRR, ontology engineering	2	Quinolone antibiotics, TCMs/COVID-19
8	Other/Unconventional AI methods	Association rule mining (ARM), MUTARC/UTARs, YOLO object detection	3	Drugs affecting cognition in CKD, broad-spectrum drugs, and unanticipated AE patterns

* Methods categorization is not mutually exclusive since several studies employed more than one particular computational or AI-based method. Therefore, the total number of studies reported in this table does not equal the total number of the included studies in this review.

After 2015, a shift towards more complex tasks of machine learning techniques emerged [29, 45–49, 51–54, 56, 57, 59–67]. Several supervised learning algorithms, such as XGBoost, Random Forest, and Support Vector Machines (SVM), were progressively utilized to support dose prediction, risk classification, and toxicity assessment [45–49, 51, 53, 54, 57, 59, 61–65]. Studies that adopted machine learning techniques reported positive findings due to its effectiveness and high accuracy in yielding reliable predictive power [29, 45–49, 51–54, 56, 57, 59–67]. These studies concluded that ML is a highly versatile method and is effective, especially in supporting personalized medicine and providing clinical decision support [29, 45–49, 51–54, 56, 57, 59–67].

Deep learning (DL) models and Natural Language Processing (NLP) have also been increasingly employed in pharmacovigilance recently, especially transformer-based NLP such as Bidirectional Encoder Representations from Transformers (BERT) [28, 33–36, 41, 43–45, 50, 70–81]. Deep learning models like Bidirectional Long Short-Term Memory (BiLSTM) or Convolutional Neural Networks (CNNs) were more prevalently used in time-series prediction or image-based diagnosis [45, 70, 71, 75]. A growing trend of using deep learning models in multi-source data or complex diseases such as Parkinson’s was shown [25] NLP, meanwhile, is more popular for being widely used for extracting ADRs from unstructured data such as summaries, social media, and package inserts [28, 33–36, 41, 43, 44, 73, 76–81]. It is often combined with ML methods for better classification or signal detection [81]. Several studies reported favourable extraction or classification performance when applying NLP to unstructured text, including spontaneous reports, social-media data, package inserts, and clinical narratives. Evidence that these improvements translated into better post-marketing safety decisions was limited [28, 33–36, 41, 43, 44, 73, 76–79, 81]. Apart from ML-NLP approach combinations, the use of general neural network methods in pharmacovigilance is often in conjunction with ML, DL, or NLP contexts, indicating a trend towards a hybrid approach in pharmacovigilance research [45, 47, 57, 59, 65].

Ontology and knowledge-based approaches were examined in several recent studies. Explanation techniques were also applied to selected machine-learning models to make individual predictions or feature contributions more interpretable [28, 49, 55, 82]. Symbolic AI was more commonly used in structured data, such as medical products, with emphasis on interpretability and traceability [28]. XAI, meanwhile, appeared to be used in acute coronary syndrome prediction and vaccine risk monitoring [49, 55]. The studies reported that explanation techniques could identify features contributing to model predictions. However, the included evidence did not establish whether these explanations improved clinician understanding, regulatory acceptance, or decision quality. Lastly, other AI methods such as ARM, MUTARC/UTARs, and YOLO were reported to be used in rare signal discovery and complex association mining. The studies highlighted their powerful capability in hypothesis generation or exploratory analysis [32, 40, 83].

### Challenges in AI application in pharmacovigilance

Notwithstanding the positive outcomes of AI applications in pharmacovigilance, the reviewed studies also highlighted several key challenges (Table 2) of AI use in pharmacovigilance systems below:

**TABLE 2:** Challenges and Recommendations on AI integration in Pharmacovigilance in Asia-Pacific (Asia-Pacific Systematic Review, 2026).

Challenge Area	Key Issues	Recommended Solutions
Data quality and generalizability (n = 24)	• Poor data quality and sparsity • Inconsistent datasets • Unstructured narrative reports • Fragmented data silos • Lack of interoperability	Enhance data integration & diversity (n = 9) • Expand multi-center data integration • Include patient-generated and social media data • Incorporate heterogeneous structured/unstructured data
Clinical integration (n = 18)	• Lack of digital maturity • Manual reporting limitations • Legal/procedural data sharing issues • Poor AI output interpretability • Validation gaps with real-world outcomes	Promote clinical validation & regulatory alignment (n = 15) • Deploy models in real-world clinical settings • Integrate into regulatory workflows • Apply AI in early drug development and post-marketing surveillance • Use explainable AI (XAI) models
Technical implementation (n = 15)	• Complex medical terminology handling • Ineffective rare ADR detection • Class imbalance issues • High computational demands • Black-box model explainability problems	Improve AI model robustness & techniques (n = 11) • Implement advanced ML techniques (GANs, SMOTE) • Use specialized deep learning networks • Enhance preprocessing and algorithm tuning • Continuously update dictionaries
Regional & cultural adaptation (=8)	• Regional language nuances • TCM complexity and non-standardized formats • Lack of digital infrastructure • Regulatory fragmentation • Cultural bias in self-reported data	Address cultural & educational gaps (n = 10) • Improve language models with domain-specific data • Tailor AI models for region-specific contexts • Provide education/training for healthcare professionals

#### Data quality and standardization challenges

Several studies recorded inadequacy of data quality, sparsity, and inconsistent or incomplete datasets hindering the performance of AI algorithms during the model training process [28, 44, 45, 48, 58]. Two studies reported difficulties during the learning process due to limited dataset annotation and low inter-annotator agreement [76, 78]. In addition to this, the use of unstructured and informal language in narrative reports further added complexity in the accuracy of the data extraction process as well as data standardization [33, 34, 43, 79]. These studies identified poor semantic consistency in datasets sourced from social media or spontaneous reports [43, 80, 84]. Furthermore, other studies [51, 55, 67] highlight challenges in the harmonization and integration of heterogeneous data sources due to fragmented data silos, a lack of interoperability between reporting systems, and discrepancies in relevant regulations.

#### Limited generalizability

Lack of geographic diversity and pharmacogenetic variation were reported by some studies as restricting model generalizability to broader populations or clinical contexts [29, 53, 54, 57, 77]. This issue is particularly prevalent in single-center studies [49, 52, 65], where model transferability to broader demographic scopes and healthcare practices was restricted due to the nature of the narrowed scope of the data. Additionally, the use of AI models in traditional medicines such as TCM and Kampo poses more challenges since language and cultural differences exist to add difficulties in cross-contextual generalization.

#### Integration with clinical workflows and health systems

Several studies identified barriers that could hinder the future deployment or operational use of AI-based methods, including limited digital infrastructure, poor interoperability, and the absence of standardised implementation protocols [32, 48, 56, 64, 69, 75, 82, 83]. The reliance on manual or semi-structured reporting limits the incorporation of AI methods in clinical settings, as reported by studies conducted in Japan and Singapore [66, 73]. Additionally, Kittisorayut et al [83] and Noguchi [32] noted that non-uniform data formats and limited integration planning were identified as barriers to translating model performance into clinical or pharmacovigilance workflows. Some other studies also find barriers in the interpretability of AI outputs, where AI-generated analytics face scrutiny from clinicians due to a lack of rationale or clinical context [32, 42, 82]. Lastly, another challenge is relevant to validation gaps between AI predictions and clinical outcomes in real-world settings [29, 85], especially in AI models developed with single-center data [52, 53], excluding key variables such as pharmacogenetics, comorbidities, and co-medications [48, 86]. This failure to account for those clinical factors in pharmacovigilance may limit the study’s generalizability to wider patient populations [46, 60].

#### Technical implementation barriers

Numerous studies in this review cited substantial issues related to design, training, and computational efficiency. The process of automated information extraction, for example, was noted in two studies as being hindered by the issues with handling complex medical terminology and out-of-vocabulary terms [35] as well as the dependence on manual feature engineering [70]. In addition to this, ineffectiveness in detecting rare ADRs was reported in some studies [40, 74], often particularly due to class imbalance and limited data variability [47, 50, 59]. Other studies [27, 32, 48] also underline the model complexity of computational demands, particularly in integrating temporal and clinical variables or modeling multifactorial ADRs [58]. In traditional signal detection models such as BCPNN, a lack of sensitivity in low-report scenarios was identified with the possibility of missing rare but critical ADRs [30, 39]. Lastly, the application of black-box model designs in pharmacovigilance, such as deep learning models, remains an issue since explainability and transparency are critical in safety assessments and regulatory validation [42, 50, 82].

#### Regional and cultural adaptation challenges

The nuances of regional languages, such as Chinese semantics and Korean narrative structures, were reported in two studies as introducing distinctive challenges to the application of NLP models [77, 86]. Some other linguistically relevant issues, such as inaccurate translation and dependence on human verification, were also noted as adding further barriers to applying AI models in multilingual settings [41]. In terms of cultural context, the distinct data structures and pharmacological profiles of TCM introduce unique challenges in AI applications. One study suggests that ingredient complexity and non-standardized reporting formats in TCM lead to ineffective ADR signal detection, especially in those AI models trained with Western pharmacovigilance data [72]. Furthermore, the lack of digital infrastructure in certain countries [69], along with regulatory fragmentation, constrains region-wide deployment of unified AI frameworks. Finally, concerns have been raised regarding data representativeness, fairness, and trust in AI-based pharmacovigilance since cultural variability and demographic bias may influence ADR data recorded through social media or other self-reported platforms [66, 80, 84].

### Recommendations for AI application in pharmacovigilance

In response to those barriers, the included studies in this review suggested the following recommendations to optimize AI applications in pharmacovigilance systems (Table 2):

#### Enhance data integration and diversity

To improve the applicability and accuracy of AI models used in pharmacovigilance systems, several studies highlighted the importance of expanding data integration across diverse datasets [46, 51, 52, 84, 87]. These studies advocated incorporating data across multiple pharmacovigilance centers, variables, and real-world sources such as patient-generated data and social media to broaden representation and generalizability beyond a single institution or population. Additionally, some other studies also emphasized the significance of including heterogeneous data, such as structured and unstructured data, to improve ADR identification and surveillance capabilities [33, 36, 43, 55].

#### Improve AI model robustness and techniques

Several studies recommended improving model robustness through methods suited to the specific data limitation or pharmacovigilance task. Suggested approaches included temporal modelling, semi-supervised learning, and resampling or synthetic-data techniques such as SMOTE and generative adversarial networks. These approaches may address particular data limitations but require careful validation because synthetic or resampled data can also introduce artefacts, amplify bias, or produce overoptimistic performance estimates [58, 59, 64, 71]. The implementation of specialized models, such as deep learning networks, and the refinement of the existing algorithms were mentioned in some studies as key recommendations for better signal detection and entity recognition [61, 70, 72, 88]. Other technique-related recommendations for building high-performing AI systems to support critical pharmacovigilance functions included enhancing preprocessing systems, tuning algorithms, and continuously updating dictionaries to ensure performance consistency [45, 76, 79].

#### Promote clinical validation and practical implementation

To ensure translational impacts of AI models in pharmacovigilance systems, many studies recommended deploying the models into the real-world settings through integration into clinical practice and regulatory workflows [48, 49, 56, 65, 89]. Several studies that used real-world data to monitor drug induced liver injury (DILI), methotrexate toxicity, or individualized tacrolimus dosing have revealed the potential effectiveness of AI models in improving risk prediction which may support therapeutic safety if the models were well integrated into clinical judgment [29, 47, 50, 60]. Furthermore, employing AI models in the early stage of drug development and post-marketing surveillance was suggested to improve ADR detection [74].

#### Facilitate interpretability and regulatory alignment

Several studies emphasised the need for interpretable and auditable outputs to support clinical review and regulatory scrutiny. Explanation techniques may assist users in examining model predictions, but they should be accompanied by documentation of data provenance, intended use, model limitations, validation populations, decision thresholds, and model updates [54, 55, 67]. Explainability should not be treated as equivalent to transparency or accountability. Responsibility for reviewing, rejecting, escalating, and acting on AI-generated safety signals must remain identifiable. This transparency process was discussed as a critical element in successful AI applications within pharmacovigilance systems. Some other studies recommended the incorporation of domain-specific ontologies, such as MedDRA, and alignment of AI models with regulatory agencies such as Pharmaceuticals and Medical Devices Agency (PMDA) to identify safety monitoring and compliance [59, 66, 89]. These recommendations are critical for both supporting end-user trust and ensuring that AI systems in pharmacovigilance align well with ethical and legal standards.

#### Addressing cultural, language, and educational gap

Owing to the contextual challenges found in each country, some studies recognized the pivotal roles of improving linguistic and cultural influence in AI applications [25, 35, 41, 43, 44, 57]. In addition to improving language models with annotated domain-specific data, these studies suggested human validation while applying machine translation systems to ensure systemic accuracy [25]. With regards to AI application in traditional medicines, such as Kampo and TCM, some studies recommended tailoring AI models by taking into account the region-specific narratives and medications [33, 72, 73, 86]. Lastly, in order to address the implementation gaps and promote system adoption, three studies highlighted the roles of education and training about ADRs, hepatotoxicity, and AI tools among healthcare professionals [35, 44, 57].

## Discussion

Our review found that published evidence on AI-based and computational pharmacovigilance methods was concentrated in a small number of Asia-Pacific countries. Japan and China accounted for half of the included studies, while only 14 of the 53 UN ESCAP members and associate members were represented. This concentration may reflect differences in pharmacovigilance infrastructure, digital-health capacity, research funding, English-language publication, and database indexing in the region. Countries with well-established pharmacovigilance systems and regulatory agencies, such as Japan through the Pharmaceuticals and Medical Devices Agency (PMDA), may have enabling environments for the development and evaluation of computational and AI-based methods [90]. In contrast, many other countries with more limited resources may still be strengthening their core fundamental pharmacovigilance functions, including regulatory governance, spontaneous reporting systems and workforce capacity, before the large-scale implementation of AI-based approaches becomes feasible. This result suggests the need for tailored implementation strategies aligned with countries' pharmacovigilance readiness and regulatory capacities rather than assuming a uniform pathway for AI adoption.

This review complements earlier global reviews of AI in pharmacovigilance. Salas et al. [16] primarily examined the types of AI methods used, their performance, and technical barriers across an international literature base. The present review identifies several issues that are especially salient in the Asia-Pacific evidence, including multilingual text processing, pharmacovigilance for traditional medicines, heterogeneous reporting systems, uneven digital infrastructure, and the concentration of research in a limited number of countries. However, these regional observations should be interpreted cautiously because the search was restricted to English-language publications and did not capture evidence from most Asia-Pacific countries.

The apparent technical promise of the reviewed models should be interpreted in light of their validation limitations. Although many studies reported favourable discrimination, classification, or information-extraction results, 11 of the 14 prediction-model studies assessed with PROBAST had a high overall risk of bias. Common limitations included absent external validation, inadequate assessment of model optimism, and insufficient reporting of missing-data handling. Consequently, the available evidence supports the feasibility of model development more strongly than it supports model transportability, clinical utility, or improvement in patient-safety outcomes.

Our study identified substantial challenges of AI integration in the Asia-Pacific landscape. Lack of standardized annotation and other data quality issues keep hindering the process of model learning and evaluation, especially in unstructured ADR reports with minimal inter-annotator agreement. Those models developed in single-center studies often face issues in generalizability and transferability owing to genetic, geographic, and cultural heterogeneity. This result corroborates the previous study conducted by Biswas [91], which found that the diversity of geographical, cultural and medical practices consistently hinders proactive pharmacovigilance practices in Asia. Moreover, this review also found that poor digital infrastructures, fragmented data formats, and limited interpretability add to the complexity of AI system adoption and integration in the healthcare system in the Asia-Pacific. Technical medical language, limited data variability, and heavy computational demands were highlighted as key technical barriers hampering AI performance in pharmacovigilance systems. Lastly, several unique challenges in the Asia-Pacific, such as linguistic diversity, socio-economic levels and traditional medicine complexity, emerge and impede an integrated AI framework in the region. Collectively, these challenges -ranging from healthcare infrastructure to technical barriers and culture complexities-reflect both systemic and technological limitations that vary across the countries in the Asia-Pacific region, suggesting the need for harmonized, context-specific efforts to achieve successful AI-PV implementation.

The included studies discussed interpretability, representativeness, and validation, but provided limited direct analysis of broader governance issues. Drawing on these findings and international guidance on AI for health, several additional considerations concerning accountability, bias, transparency, and human oversight warrant discussion. The WHO report highlights several ethical issues that should become our primary attention regarding AI use in healthcare, including accountability, contextual bias and transparency [92]. Accountability and liability present complex challenges that are further magnified in the Asia-Pacific region, where many low- and middle-income countries lack robust, specific regulatory frameworks for AI in healthcare. Without clear legal and regulatory oversight, there is a significant risk that liability will be unfairly shifted onto local clinicians or health systems, while international developers may evade accountability. Addressing these ethical dimensions requires not only the technical refinement of AI models but also the urgent establishment of clear, region-if not-country-specific governance and liability frameworks that are aligned with global standards. The issue of bias from the current AI data sources is also prominent for the region as current AI models trained on geographically, ethnically, or socioeconomically unrepresentative datasets—risking unequal ADR detection performance for populations in the Asia Pacific. This bias will directly contravene the WHO principle of ensuring inclusiveness and equity in AI deployment. Transparency is equally critical, particularly regarding the integration of AI into clinical workflows and health systems. High-performing deep learning models often operate as “black boxes,” obscuring the rationale behind generated safety signals. In the Asia-Pacific context, as this is compounded by contextual bias—Asia-Pacific clinicians are presented with unexplainable safety signals derived from foreign populations that do not reflect local genetic, epidemiological, or prescribing realities, their trust in the AI system’s clinical relevance may further erode. Consequently, healthcare providers may dismiss these AI-generated recommendations as contextually inappropriate or irrelevant to their patients [93]. To maintain trust and ensure human autonomy, AI tools must not only be intelligible—allowing clinicians to interrogate and override recommendations—but they must also be transparently validated using representative Asia Pacific data to prove their precision in the real world clinical setting. Human oversight should be specified as an operational process rather than stated only as a general principle. AI-generated signals require defined procedures for review, escalation, rejection, documentation, and override. Post-deployment monitoring should assess model drift, false-positive burden, subgroup performance, automation bias, and alert fatigue. The responsible human decision-maker should remain identifiable throughout the signal-management process.

We also want to outline several recommendations proposed by the included studies. To improve generalizability and representation, several studies suggested improving data integration through the involvement of diverse, multi-institutional, and real-world data sources [46, 51, 52, 84, 87]. Certain advanced techniques, such as temporal modeling, semi-supervised learning, and continual algorithm adjustment, were recommended to improve model robustness. In terms of practical utility, many studies highlighted the significance of real-world clinical validation, integration into regulatory workflows, and deployment across drug development stages [48, 49, 56, 65, 89]. The application of explainable AI (XAI) models and system alignment with standards like MedDRA were noted as essential for facilitating interpretability and fostering clinical trust. This growing recognition of XAI models and MedDRA standards reflects on the importance of balancing sophistication with transparency to achieve wider adoption, as also supported by the WHO report in ethics and governance of artificial intelligence for health [92]. In addition to that, addressing linguistic, cultural, and educational gaps were deemed as crucial approaches to support region-wide adoption of AI frameworks in the Asia-Pacific. This educational approach has been highlighted in some studies conducted in low- and middle-income countries [94, 95], which found that enhancing healthcare professionals’ knowledge and skills in pharmacovigilance is a key strategy for strengthening pharmacovigilance systems.

Translating these recommendations into practice may require a capability-based rather than algorithm-based approach. Countries or institutions with limited digital infrastructure may initially prioritise standardised adverse-event reporting, data quality, interoperable terminologies, workforce development, and basic analytic capacity. Settings with stronger data and governance capabilities may additionally evaluate externally validated machine-learning or natural-language-processing systems, provided that appropriate human oversight, auditability, and post-deployment monitoring are in place. The appropriate method should be determined by the pharmacovigilance task, available data, validation evidence, and regulatory requirements rather than by an assumption that more complex algorithms represent a more mature system. This proposed approach is an interpretation derived from the review and requires prospective evaluation.

### Limitations

First, search and temporal constraints may limit our review comprehensiveness. Restricting searches to MEDLINE, Scopus, and the top 100 English-language Google Scholar results may have excluded regional, non-English, or engineering-focused literature.

Second, geographic and contextual factors restrict generalizability. The evidence is heavily concentrated in Japan and China (representing only 14 of 53 UN ESCAP members). Furthermore, because some studies addressed broader clinical prediction rather than routine pharmacovigilance, the findings reflect computational method development more than operational implementation. Consequently, publications analyzed in our review indicate regional research activity rather than direct evidence of each country’s AI readiness or regulatory maturity.

Third, methodological heterogeneity precluded quantitative synthesis and standardized quality appraisal. Diverse study designs necessitated a descriptive, non-validated risk-of-bias assessment, which the current available tools might not be able to quantify correctly.

### Conclusion

This review identified the use of AI-based and conventional computational methods for pharmacovigilance analysis in the Asia-Pacific. To support the effective implementation of AI-based pharmacovigilance in the region, a tiered implementation strategy can be employed through establishing a regional collaboration framework and taking into account disparities in technological maturity across countries in the region.

## References

[1] Euromonitor International. Pharmaceuticals and Medical Equipment in Asia Pacific (2022). Available online at: https://lp.euromonitor.com/pharmaceuticals-and-medical-equipment-in-asia-pacific/report (Accessed August 13, 2025).

[2] Garashi HY Steinke DT Schafheutle EI . A systematic review of pharmacovigilance systems in developing countries using the WHO pharmacovigilance indicators. Ther Innov Regul Sci (2022) 56(5):717–43. 10.1007/s43441-022-00415-y PMC9356965 35657484

[3] World Health Organization. Resolutions – patient safety policy (2026). Available online at: https://www.who.int/teams/integrated-health-services/patient-safety/policy/resolutions (Accessed August 5, 2025).

[4] Putri RA Ikawati Z Rahmawati F Yasin NM . An awareness of pharmacovigilance among healthcare professionals dueto an underreporting of adverse drug reactions issue: a SystematicReview of the current state, obstacles, and strategy. Curr Drug Saf (2024) 19(3):317–31. 10.2174/0115748863276456231016062628 PMC11327747 38989832

[5] N IO Kp S R JR Mg R . Barriers to and facilitators of healthcare professionals in ADR reporting in a tertiary care hospital in India. BMC Health Serv Res (2025) 25(1):166. 10.1186/s12913-024-12139-w 39875957 PMC11773872

[6] Endale BS Wallelign TM Gedif Fenta T . Implementation status and challenges of pharmacovigilance program in Ethiopia: a mixed-methods study. Inq J Health Care Organ Provis Financ (2024) 61:00469580241287752. 10.1177/00469580241287752

[7] Hartigan-Go K . Developing a pharmacovigilance system in the Philippines, a country of diverse culture and strong traditional medicine background. Toxicology (2002) 181-182:103–7. 10.1016/S0300-483X(02)00263-9 12505293

[8] World Health Organization. Pharmacovigilance and traditional and complementary medicine in south-east Asia: a situation review (2019). Available online at: https://www.who.int/publications/i/item/9789290227250 (Accessed July 29, 2025).

[9] Singh S Pamnani I Rang S Malik R Punitha A Purkait R . Pharmacovigilance initiative for ayush drugs in India. Int J Ayurveda Res (2023) 4(2):102–6. 10.4103/ijar.ijar_12_23

[10] World Health Organization. The pharmacovigilance system for traditional medicine in Thailand (2017). Available online at: https://www.who.int/publications/i/item/sea-hsd-393 (Accessed July 26, 2025).

[11] Shimada Y Fujimoto M Nogami T Watari H Kitahara H Misawa H Patient safety incident reports related to traditional Japanese kampo medicines: medication errors and adverse drug events in a university hospital for a ten-year period. BMC Complement Altern Med (2017) 17(1):547. 10.1186/s12906-017-2051-2 29268743 PMC5740942

[12] Zhang L Yan J Liu X Ye Z Yang X Meyboom R Pharmacovigilance practice and risk control of traditional Chinese medicine drugs in China: current status and future perspective. J Ethnopharmacol (2012) 140(3):519–25. 10.1016/j.jep.2012.01.058 22374080

[13] Withers DR . Traditional medicine and modern pharmacovigilance: challenges and opportunities. J Pharmacovigil (2023) 11(4):1000444. 10.35248/2329-6887.23.11.444

[14] Chindhalore CA Mohod B Gajbhiye S Dakhale GN Dhal S . Exploring artificial intelligence integration in Indian pharmacology: a survey on scope, threats, and challenges. Ann Afr Med (2025) 25:144–50. 10.4103/aam.aam_59_25 PMC12872159 40452317

[15] Song H Pei X Liu Z Shen C Sun J Liu Y Pharmacovigilance in China: evolution and future challenges. Br J Clin Pharmacol (2023) 89(2):510–22. 10.1111/bcp.15277 PMC10078654 35165914

[16] Salas M Petracek J Yalamanchili P Aimer O Kasthuril D Dhingra S The use of artificial intelligence in pharmacovigilance: a systematic review of the literature. Pharm Med (2022) 36(5):295–306. 10.1007/s40290-022-00441-z 35904529

[17] Schmider J Kumar K LaForest C Swankoski B Naim K Caubel PM . Innovation in pharmacovigilance: use of artificial intelligence in adverse event case processing. Clin Pharmacol Ther (2019) 105(4):954–61. 10.1002/cpt.1255 30303528 PMC6590385

[18] Dimitsaki S Natsiavas P Jaulent MC . Applying AI to structured real-world data for pharmacovigilance purposes: scoping review. J Med Internet Res (2024) 26:e57824. 10.2196/57824 PMC11729787 39753222

[19] Dsouza VS Leyens L Kurian JR Brand A Brand H . Artificial intelligence (AI) in pharmacovigilance: a systematic review on predicting adverse drug reactions (ADR) in hospitalized patients. Res Soc Adm Pharm (2025) 21(6):453–62. 10.1016/j.sapharm.2025.02.008 39961738

[20] Sallam M Snygg J Sallam M . Can google scholar serve as a sufficient source for systematic reviews? Reconsidering the need for multiple database searches. Weblog J Med Sci (2026) V2(1). 10.5281/zenodo.19764716

[21] Page MJ McKenzie JE Bossuyt PM Boutron I Hoffmann TC Mulrow CD The PRISMA 2020 statement: an updated guideline for reporting systematic reviews. BMJ (2021) 372(n71). 10.1136/bmj.n71 PMC8005924 33782057

[22] Economic and Social Commission for Asia and the Pacific. ESCAP Members and Associate Members (2026). Available online at: https://www.unescap.org/about/member-states (Accessed June 3, 2025).

[23] Langerhuizen DWG Janssen SJ Mallee WH van den Bekerom MPJ Ring D Kerkhoffs GMMJ What are the applications and limitations of artificial intelligence for fracture detection and classification in orthopaedic trauma imaging? A systematic review. Clin Orthop (2019) 477(11):2482–91. 10.1097/CORR.0000000000000848 31283727 PMC6903838

[24] Groot OQ Bongers MER Ogink PT Senders JT Karhade AV Bramer JAM Does artificial intelligence outperform natural intelligence in interpreting musculoskeletal radiological studies? A systematic review. Clin Orthop (2020) 478(12):2751–64. 10.1097/CORR.0000000000001360 32740477 PMC7899420

[25] Ye Q Yuan XL Yuan CX Zhang HZ Yang XM . Zishenpingchan granules for the treatment of Parkinson’s disease: a randomized, double-blind, placebo-controlled clinical trial. Neural Regen Res (2018) 13(7):1269–75. 10.4103/1673-5374.235075 30028337 PMC6065246

[26] Joanna Briggs Institute. Critical Appraisal Tools (2026). Available online at: https://jbi.global/critical-appraisal-tools (Accessed April 6, 2026).

[27] Ye X Fu Z Wang H Du W Wang R Sun Y A computerized system for signal detection in spontaneous reporting system of shanghai China. Pharmacoepidemiol Drug Saf (2009) 18(2):154–8. 10.1002/pds.1695 19115240

[28] Li X Lin X Ren H Guo J . Ontological organization and bioinformatic analysis of adverse drug reactions from package inserts: development and usability study. J Med Internet Res (2020) 22(7):e20443. 10.2196/20443 32706718 PMC7400033

[29] Zeng Y Lu H Li S Shi QZ Liu L Gong YQ Risk prediction of liver injury in pediatric tuberculosis treatment: development of an automated machine learning model. Drug Des Devel Ther (2025) 19:239–50. 10.2147/DDDT.S495555 39830784 PMC11740905

[30] Kubota K Koide D Hirai T . Comparison of data mining methodologies using Japanese spontaneous reports. Pharmacoepidemiol Drug Saf (2004) 13(6):387–94. 10.1002/pds.964 15170768

[31] Nomura K Takahashi K Hinomura Y Kawaguchi G Matsushita Y Marui H Effect of database profile variation on drug safety assessment: an analysis of spontaneous adverse event reports of Japanese cases. Drug Des Devel Ther (2015) 9:3031–41. 10.2147/DDDT.S81998 26109846 PMC4472069

[32] Noguchi Y Nagasawa H Tachi T Tsuchiya T Teramachi H . Signal detection of oral drug-induced dementia in chronic kidney disease patients using association rule mining and Bayesian confidence propagation neural network. Pharmazie (2019) 74(9):570–4. 10.1691/ph.2019.9426 31484600

[33] Matsuda S Ohtomo T Tomizawa S Miyano Y Mogi M Kuriki H Incorporating unstructured patient narratives and health insurance claims data in pharmacovigilance: natural language processing analysis of patient-generated texts about systemic lupus erythematosus. JMIR Public Health Surveill (2021) 7(6):e29238. 10.2196/29238 PMC8278300 34255719

[34] Mashima Y Tamura T Kunikata J Tada S Yamada A Tanigawa M Using natural language processing techniques to detect adverse events from progress notes due to chemotherapy. Cancer Inform (2022) 21:11769351221085064. 10.1177/11769351221085064 PMC8943584 35342285

[35] Maeda-Minami A Yoshino T Yumoto T Sato K Sagara A Inaba K Development of a novel drug information provision system for kampo medicine using natural language processing technology. BMC Med Inform Decis Mak (2023) 23(1):119. 10.1186/s12911-023-02230-3 PMC10347708 37442993

[36] Yada S Nishiyama T Wakamiya S Kawazoe Y Imai S Hori S Utility analysis and demonstration of real-world clinical texts: a case study on Japanese cancer-related EHRs. PLoS ONE (2024) 19(9):e0310432. 10.1371/journal.pone.0310432 PMC11389901 39259727

[37] Lou S Cui Z Ou Y Chen J Zhou L Zhao R A multidimensional assessment of adverse events associated with paliperidone palmitate: a real-world pharmacovigilance study using the FAERS and JADER databases. BMC Psychiatry (2025) 25(1):52. 10.1186/s12888-025-06493-0 39833706 PMC11744949

[38] Guan Y Qi Y Zheng L Yang J Zhang M Zhang Q Data mining techniques for detecting signals of adverse drug reaction of cardiac therapy drugs based on jinan adverse event reporting system database: a retrospective study. BMJ Open (2023) 13(1):e068127. 10.1136/bmjopen-2022-068127 36669842 PMC9872458

[39] Ang PS Chen Z Chan CL Tai BC . Data mining spontaneous adverse drug event reports for safety signals in Singapore–a comparison of three different disproportionality measures. Expert Opin Drug Saf (2016) 15(5):583–90. 10.1517/14740338.2016.1167184 26996192

[40] Jin H Chen J He H Williams GJ Kelman C O’Keefe CM . Mining unexpected temporal associations: applications in detecting adverse drug reactions. IEEE Trans Inf Technol Biomed (2008) 12(4):488–500. 10.1109/TITB.2007.900808 18632329

[41] Wong E Symons K . The australian injectable drugs handbook (AIDH): experience of artificial intelligence translations of Non-English product information. J Pharm Pract Res (2024) 55:68–78. Published online. 10.1002/jppr.1946

[42] Modayil RR Harugeri A Parthasarathi G Ramesh M Prasad R Naik V Adverse drug reactions to antiretroviral therapy (ART): an experience of spontaneous reporting and intensive monitoring from ART centre in India. Pharmacoepidemiol Drug Saf (2010) 19(3):247–55. 10.1002/pds.1907 20066675

[43] Rajalakshmi P Rajagopalan SP . Sentiment ontology analysis for adverse drug effect. Medico-Leg Update (2019) 19(2):280–5. 10.5958/0974-1283.2019.00188.9

[44] Gurudiwan P Sharma P . The ability and tasks of pharmacovigilance and adverse drug events in India a global scenario. South East Eur J Public Health (2024) 23(2):297–301. 10.70135/seejph.vi.790

[45] Vu HD Pham VH Le QD . Drug classification system based on drug composition and usage instructions. EAI Endorsed Trans Ind Netw Intell Syst (2025) 12(1):1–8. 10.4108/EETINIS.V12I1.5995

[46] Guo W Yu Z Gao Y Lan X Zang Y Yu P A machine learning model to predict risperidone active moiety concentration based on initial therapeutic drug monitoring. Front Psychiatry (2021) 12:711868. 10.3389/fpsyt.2021.711868 PMC8637165 34867511

[47] Hu Q Wang H Xu T . Predicting hepatotoxicity associated with low-dose methotrexate using machine learning. J Clin Med (2023) 12(4):1599. 10.3390/jcm12041599 PMC9967588 36836131

[48] Yalçın N Kaşıkcı M Çelik HT Allegaert K Demirkan K Yiğit Ş Development and validation of a machine learning-based detection system to improve precision screening for medication errors in the neonatal intensive care unit. Front Pharmacol (2023) 14:1151560. 10.3389/fphar.2023.1151560 37124199 PMC10140576

[49] Kim Y Jang JH Park N Jeong NY Lim E Kim S Machine learning approach for active vaccine safety monitoring. J Korean Med Sci (2021) 36(27):1–13. 10.3346/jkms.2021.36.e198 PMC8352788 34402232

[50] Heo S Yu JY Kang EA Shin H Ryu K Kim C Corrigendum: development and verification of time-series deep learning for drug-induced liver injury detection in patients taking angiotensin II receptor blockers: a multicenter distributed research network approach. Healthc Inform Res (2024) 30(2):168. 10.4258/hir.2024.30.2.168 38755108 PMC11098767

[51] Park SJ Yang S Lee S Joo SH Park T Kim DH Machine-learning parsimonious prediction model for diagnostic screening of severe hematological adverse events in cancer patients treated with PD-1/PD-L1 inhibitors: retrospective observational study by using the common data model. Diagnostics (2025) 15(2):226. 10.3390/diagnostics15020226 PMC11763827 39857110

[52] Hu YH Wu F Lo CL Tai CT . Predicting warfarin dosage from clinical data: a supervised learning approach. Artif Intell Med (2012) 56(1):27–34. 10.1016/j.artmed.2012.04.001 22537823

[53] Hu YH Tai CT Tsai CF Huang MW . Improvement of adequate digoxin dosage: an application of machine learning approach. J Healthc Eng (2018) 2018:3948245. 10.1155/2018/3948245 30210752 PMC6120286

[54] Tai CT Sue KL Hu YH . Machine learning in high-alert medication treatment: a study on the cardiovascular drug. Appl Sci Switz (2020) 10(17):5798. 10.3390/app10175798

[55] Ward IR Wang L Lu J Bennamoun M Dwivedi G Sanfilippo FM . Explainable artificial intelligence for pharmacovigilance: what features are important when predicting adverse outcomes? Explainable artificial intelligence for pharmacovigilance. Comput Methods Programs Biomed (2021) 212:106415. 10.1016/j.cmpb.2021.106415 34715520

[56] Henry BJ Lim WH Syed Ahmad SM Menon Premakumar C Mohd Tahir NA Mhd Ali A Machine learning-based risk prediction model for medication administration errors in neonatal intensive care units: a prospective direct observational study. Digit Health (2024) 10:20552076241286434. 10.1177/20552076241286434 39430694 PMC11489987

[57] Perwitasari DA Faridah IN Dania H Setiawan D Safaria T . Machine learning model to predict the adherence of tuberculosis patients experiencing increased levels of liver enzymes in Indonesia. PLoS ONE (2025) 20(1 January):e0315912. 10.1371/journal.pone.0315912 39854377 PMC11760577

[58] Hsu W Warren JR Riddle PJ . Medication adherence prediction through temporal modelling in cardiovascular disease management. BMC Med Inform Decis Mak (2022) 22(1):313. 10.1186/s12911-022-02052-9 36447245 PMC9710081

[59] Wei J Feng G Lu Z Han P Zhu Y Huang W . Evaluating drug risk using GAN and SMOTE based on cFDA’s spontaneous reporting data. J Healthc Eng (2021) 2021:1–14. 10.1155/2021/6033860 PMC8418931 34493954

[60] Fu Q Jing Y Liu Mr G Jiang Mr X Liu H Kong Y Machine learning-based method for tacrolimus dose predictions in Chinese kidney transplant perioperative patients. J Clin Pharm Ther (2022) 47(5):600–8. 10.1111/jcpt.13579 34802160

[61] Watanabe T Ambe K Tohkin M . Predicting the addition of information regarding clinically significant adverse drug reactions to Japanese drug package inserts using a machine-learning model. Ther Innov Regul Sci (2024) 58(2):357–67. 10.1007/s43441-023-00603-4 38135862 PMC10850196

[62] Enokiya T Ozaki K . Developing an AI-based prediction model for anaphylactic shock from injection drugs using Japanese real-world data and chemical structure-based analysis. DARU J Pharm Sci (2024) 32(1):253–62. 10.1007/s40199-024-00511-4 PMC11087410 38580799

[63] Lee JE Kim JH Bae JH Song I Shin JY . Detecting early safety signals of infliximab using machine learning algorithms in the Korea adverse event reporting system. Sci Rep (2022) 12(1):14869. 10.1038/s41598-022-18522-z PMC9436954 36050484

[64] Jang MG Cha S Kim S Lee S Lee KE Shin KH . Application of tree-based machine learning classification methods to detect signals of fluoroquinolones using the Korea adverse event reporting system (KAERS) database. Expert Opin Drug Saf (2023) 22(7):629–36. 10.1080/14740338.2023.2181341 36794497

[65] Lai NH Shen WC Lee CN Chang JC Hsu MC Kuo LN Comparison of the predictive outcomes for anti-tuberculosis drug-induced hepatotoxicity by different machine learning techniques. Comput Methods Programs Biomed (2020) 188:105307. 10.1016/j.cmpb.2019.105307 31911332

[66] Ang PS Teo DCH Dorajoo SR Prem Kumar M Chan YH Choong CT Augmenting product defect surveillance through web crawling and machine learning in Singapore. Drug Saf (2021) 44(9):939–48. 10.1007/s40264-021-01084-w 34148223 PMC8214454

[67] Choo SM Sartori D Lee SC Yang HC Syed-Abdul S . Data-driven identification of factors that influence the quality of adverse event reports: 15-year interpretable machine learning and time-series analyses of VigiBase and QUEST. JMIR Med Inform (2024) 12:e49643. 10.2196/49643 38568722 PMC11024759

[68] Li C Xia J Deng J Jiang J . A comparison of measures of disproportionality for signal detection on adverse drug reaction spontaneous reporting database of Guangdong province in China. Pharmacoepidemiol Drug Saf (2008) 17(6):593–600. 10.1002/pds.1601 18432629

[69] Rekha BH Hisham SA Wahab IA Ali NM Goh KW Ming LC . Digital monitoring of medication safety in children: an investigation of ADR signalling techniques in Malaysia. BMC Med Inform Decis Mak (2024) 24(1):395. 10.1186/s12911-024-02801-y 39695558 PMC11657009

[70] Yang H Gao H . Toward sustainable virtualized healthcare: extracting medical entities from chinese online health consultations using deep neural networks. Sustain Switz (2018) 10(9):3292. 10.3390/su10093292

[71] Chen Y Zhou C Li T Wu H Zhao X Ye K Named entity recognition from Chinese adverse drug event reports with lexical feature based BiLSTM-CRF and tri-training. J Biomed Inform (2019) 96:103252. 10.1016/j.jbi.2019.103252 31323311

[72] Wei J Dai J Sun Y Meng Z Ma H Zhou Y . TIRPnet: risk prediction of traditional Chinese medicine ingredients based on a deep neural network. J Ethnopharmacol (2024) 325:117860. 10.1016/j.jep.2024.117860 38316222

[73] Ujiie S Yada S Wakamiya S Aramaki E . Identification of adverse drug event–related japanese articles: natural language processing analysis. JMIR Med Inform (2020) 8(11):e22661. 10.2196/22661 33245290 PMC7732716

[74] Ambe K Ohya K Takada W Suzuki M Tohkin M . *In silico* approach to predict severe cutaneous adverse reactions using the Japanese adverse drug event report database. Clin Transl Sci (2021) 14(2):756–63. 10.1111/cts.12944 33417306 PMC7993315

[75] Fujimoto A Iwai Y Ishikawa T Shinkuma S Shido K Yamasaki K Deep neural network for early image diagnosis of stevens-johnson syndrome/toxic epidermal necrolysis. J Allergy Clin Immunol Pract (2022) 10(1):277–83. 10.1016/j.jaip.2021.09.014 34547536

[76] Kizaki H Satoh H Ebara S Watabe S Sawada Y Imai S Construction of a multi-label classifier for extracting multiple incident factors from medication incident reports in residential care facilities: natural language processing approach. JMIR Med Inform (2024) 12:e58141. 10.2196/58141 39042454 PMC11303886

[77] Kim S Kang T Chung TK Choi Y Hong Y Jung K Automatic extraction of comprehensive drug safety information from adverse drug event narratives in the Korea adverse event reporting system using natural language processing techniques. Drug Saf (2023) 46(8):781–95. 10.1007/s40264-023-01323-2 37330415 PMC10344995

[78] Ang PS Fan LYP Tham MY Tan SH Soh SB Foo BP Towards human–machine collaboration in creating an evaluation corpus for adverse drug events in discharge summaries of electronic medical records. Big Data Res (2016) 4:37–43. 10.1016/j.bdr.2016.04.001

[79] Tang Y Yang J Ang PS Dorajoo SR Foo B Soh S Detecting adverse drug reactions in discharge summaries of electronic medical records using readpeer. Int J Med Inf (2019) 128:62–70. 10.1016/j.ijmedinf.2019.04.017 31160013

[80] Jarynowski A Semenov A Kamiński M Belik V . Mild adverse events of sputnik V vaccine in Russia: social media content analysis of telegram *via* deep learning. J Med Internet Res (2021) 23(11):e30529. 10.2196/30529 34662291 PMC8631420

[81] Ma J Chen H Sun J Huang J He G Yang G . Efficient analysis of drug interactions in liver injury: a retrospective study leveraging natural language processing and machine learning. BMC Med Res Methodol (2024) 24(1):312. 10.1186/s12874-024-02443-8 39707270 PMC11660714

[82] Wang Z Li L Song M Yan J Shi J Yao Y . Evaluating the traditional chinese medicine (TCM) officially recommended in China for COVID-19 using ontology-based side-effect prediction framework (OSPF) and deep learning. J Ethnopharmacol (2021) 272:113957. 10.1016/j.jep.2021.113957 33631276 PMC7899032

[83] Kittisorayut S Nonsiri S Kamin P Makdee S . A machine learning algorithms for drug identification: enhancing medication safety in Thailand. (2024) 47–52. 10.1109/JCSSE61278.2024.10613653

[84] Nguyen T Larsen ME O’Dea B Phung D Venkatesh S Christensen H . Estimation of the prevalence of adverse drug reactions from social media. Int J Med Inf (2017) 102:130–7. 10.1016/j.ijmedinf.2017.03.013 28495341

[85] Imai S Kadomura S Miyai T Kashiwagi H Sato Y Sugawara M Using Japanese big data to investigate novel factors and their high-risk combinations that affect vancomycin-induced nephrotoxicity. Br J Clin Pharmacol (2022) 88(7):3241–55. 10.1111/bcp.15252 35106797

[86] Feng ZY Wu XH Ma JL Li M He GF Cao DS DKADE: a novel framework based on deep learning and knowledge graph for identifying adverse drug events and related medications. Brief Bioinform (2023) 24(4):bbad228. 10.1093/bib/bbad228 37344167

[87] Watanabe T Ambe K Tohkin M . Streamlining considerations for safety measures: a predictive model for addition of clinically significant adverse reactions to Japanese drug package inserts. Biol Pharm Bull (2024) 47(3):611–9. 10.1248/bpb.b23-00846 38479885

[88] Wang CS Lin PJ Cheng CL Tai SH Kao Yang YH Chiang JH . Detecting potential adverse drug reactions using a deep neural network model. J Med Internet Res (2019) 21(2):e11016. 10.2196/11016 30724742 PMC6381404

[89] Okamoto R Kojima R Nakatsui M . Toward AI-supported evaluation for safety control measures against near-miss events in pharmaceutical products. Saf Sci (2023) 168:106314. 10.1016/j.ssci.2023.106314

[90] Pharmaceuticals and medical devices agency. Pharmaceuticals and Medical Devices Agency (PMDA) (2026). Available online at: https://www.pmda.go.jp/english/(Accessed August 3, 2026).

[91] Biswas P . Pharmacovigilance in Asia. J Pharmacol Pharmacother (2013) 4(1_Suppl. l):S7–S19. 10.4103/0976-500X.120941 24347987 PMC3853674

[92] World Health Organization. Ethics and Governance of Artificial Intelligence for Health: WHO Guidance (2021).

[93] Kelly CJ Karthikesalingam A Suleyman M Corrado G King D . Key challenges for delivering clinical impact with artificial intelligence. BMC Med (2019) 17(1):195. 10.1186/s12916-019-1426-2 31665002 PMC6821018

[94] Menang O Kuemmerle A Maigetter K Burri C . Strategies and interventions to strengthen pharmacovigilance systems in low-income and middle-income countries: a scoping review. BMJ Open (2023) 13(9):e071079. 10.1136/bmjopen-2022-071079 37709326 PMC10503375

[95] Kiguba R Olsson S Waitt C . Pharmacovigilance in low‐ and middle‐income countries: a review with particular focus on Africa. Br J Clin Pharmacol (2023) 89(2):491–509. 10.1111/bcp.15193 34937122

