# Supplementary material for: Artificial intelligence and computational methods in the Asia-Pacific pharmacovigilance landscape: a systematic review
Source: Int J Public Health. 2026 Sep 11;71:1610026. doi: 10.3389/ijph.2026.1610026 (PMC13612370; doi:10.3389/ijph.2026.1610026)
Supplement: Supplementary file 1 [file Table1.docx]

**Supplementary Files.**

**Supplementary File 1. PRISMA Checklist**

**Bridging Pharmacovigilance Gaps in Asia-Pacific Through Artificial Intelligence: A Systematic Review**

**Table S1. PRISMA Checklists**

| **Section and Topic** | **Item #** | **Checklist item** | **Location where item is reported** |
| --- | --- | --- | --- |
| **TITLE** | | |  |
| Title | 1 | Identify the report as a systematic review. | Title |
| **ABSTRACT** | | |  |
| Abstract | 2 | See the PRISMA 2020 for Abstracts checklist. | Abstract |
| **INTRODUCTION** | | |  |
| Rationale | 3 | Describe the rationale for the review in the context of existing knowledge. | Introduction |
| Objectives | 4 | Provide an explicit statement of the objective(s) or question(s) the review addresses. | Introduction |
| **METHODS** | | |  |
| Eligibility criteria | 5 | Specify the inclusion and exclusion criteria for the review and how studies were grouped for the syntheses. | Methods |
| Information sources | 6 | Specify all databases, registers, websites, organisations, reference lists and other sources searched or consulted to identify studies. Specify the date when each source was last searched or consulted. | Methods |
| Search strategy | 7 | Present the full search strategies for all databases, registers and websites, including any filters and limits used. | Supplementary File 1 |
| Selection process | 8 | Specify the methods used to decide whether a study met the inclusion criteria of the review, including how many reviewers screened each record and each report retrieved, whether they worked independently, and if applicable, details of automation tools used in the process. | Methods |
| Data collection process | 9 | Specify the methods used to collect data from reports, including how many reviewers collected data from each report, whether they worked independently, any processes for obtaining or confirming data from study investigators, and if applicable, details of automation tools used in the process. | Methods |
| Data items | 10a | List and define all outcomes for which data were sought. Specify whether all results that were compatible with each outcome domain in each study were sought (e.g. for all measures, time points, analyses), and if not, the methods used to decide which results to collect. | Methods |
|  | 10b | List and define all other variables for which data were sought (e.g. participant and intervention characteristics, funding sources). Describe any assumptions made about any missing or unclear information. | Methods |
| Study risk of bias assessment | 11 | Specify the methods used to assess risk of bias in the included studies, including details of the tool(s) used, how many reviewers assessed each study and whether they worked independently, and if applicable, details of automation tools used in the process. | Quality Assessment (Methods) |
| Effect measures | 12 | Specify for each outcome the effect measure(s) (e.g. risk ratio, mean difference) used in the synthesis or presentation of results. | - |
| Synthesis methods | 13a | Describe the processes used to decide which studies were eligible for each synthesis (e.g. tabulating the study intervention characteristics and comparing against the planned groups for each synthesis (item #5)). | - |
|  | 13b | Describe any methods required to prepare the data for presentation or synthesis, such as handling of missing summary statistics, or data conversions. | - |
|  | 13c | Describe any methods used to tabulate or visually display results of individual studies and syntheses. | Methods |
|  | 13d | Describe any methods used to synthesize results and provide a rationale for the choice(s). If meta-analysis was performed, describe the model(s), method(s) to identify the presence and extent of statistical heterogeneity, and software package(s) used. | Methods |
|  | 13e | Describe any methods used to explore possible causes of heterogeneity among study results (e.g. subgroup analysis, meta-regression). | - |
|  | 13f | Describe any sensitivity analyses conducted to assess robustness of the synthesized results. | - |
| Reporting bias assessment | 14 | Describe any methods used to assess risk of bias due to missing results in a synthesis (arising from reporting biases). | - |
| Certainty assessment | 15 | Describe any methods used to assess certainty (or confidence) in the body of evidence for an outcome. | - |
| **RESULTS** | | |  |
| Study selection | 16a | Describe the results of the search and selection process, from the number of records identified in the search to the number of studies included in the review, ideally using a flow diagram. | Study Selection Process (Results) |
|  | 16b | Cite studies that might appear to meet the inclusion criteria, but which were excluded, and explain why they were excluded. | Study Selection Process (Results) |
| Study characteristics | 17 | Cite each included study and present its characteristics. | Study Characteristics (Results) |
| Risk of bias in studies | 18 | Present assessments of risk of bias for each included study. | Study Characteristics (Results) |
| Results of individual studies | 19 | For all outcomes, present, for each study: (a) summary statistics for each group (where appropriate) and (b) an effect estimate and its precision (e.g. confidence/credible interval), ideally using structured tables or plots. | - |
| Results of syntheses | 20a | For each synthesis, briefly summarise the characteristics and risk of bias among contributing studies. | Quality Appraisal (Results) |
|  | 20b | Present results of all statistical syntheses conducted. If meta-analysis was done, present for each the summary estimate and its precision (e.g. confidence/credible interval) and measures of statistical heterogeneity. If comparing groups, describe the direction of the effect. | - |
|  | 20c | Present results of all investigations of possible causes of heterogeneity among study results. | Results |
|  | 20d | Present results of all sensitivity analyses conducted to assess the robustness of the synthesized results. | - |
| Reporting biases | 21 | Present assessments of risk of bias due to missing results (arising from reporting biases) for each synthesis assessed. | - |
| Certainty of evidence | 22 | Present assessments of certainty (or confidence) in the body of evidence for each outcome assessed. | - |
| **DISCUSSION** | | |  |
| Discussion | 23a | Provide a general interpretation of the results in the context of other evidence. | Discussion |
|  | 23b | Discuss any limitations of the evidence included in the review. | Discussion |
|  | 23c | Discuss any limitations of the review processes used. | Discussion |
|  | 23d | Discuss implications of the results for practice, policy, and future research. | Discussion |
| **OTHER INFORMATION** | | |  |
| Registration and protocol | 24a | Provide registration information for the review, including register name and registration number, or state that the review was not registered. | - |
|  | 24b | Indicate where the review protocol can be accessed, or state that a protocol was not prepared. | - |
|  | 24c | Describe and explain any amendments to information provided at registration or in the protocol. | - |
| Support | 25 | Describe sources of financial or non-financial support for the review, and the role of the funders or sponsors in the review. | Statements |
| Competing interests | 26 | Declare any competing interests of review authors. | Statements |
| Availability of data, code and other materials | 27 | Report which of the following are publicly available and where they can be found: template data collection forms; data extracted from included studies; data used for all analyses; analytic code; any other materials used in the review. | - |

*From:*  Page MJ, McKenzie JE, Bossuyt PM, Boutron I, Hoffmann TC, Mulrow CD, et al. The PRISMA 2020 statement: an updated guideline for reporting systematic reviews. BMJ 2021;372:n71. doi: 10.1136/bmj.n71. This work is licensed under CC BY 4.0. To view a copy of this license, visit <https://creativecommons.org/licenses/by/4.0/>

**Supplementary File 2. Search Strategy**

1. **MEDLINE**

(("Artificial Intelligence"[Mesh] OR "Machine Learning"[Mesh] OR "Natural Language Processing"[Mesh] OR "Deep Learning"[tiab] OR "Neural Network*"[tiab] OR "Machine learning"[tiab] OR "Artificial intelligence"[tiab] OR "AI"[tiab] OR "Natural language processing"[tiab])) AND ("Pharmacovigilance"[Mesh] OR "Drug-Related Side Effects and Adverse Reactions"[Mesh] OR "Drug Monitoring"[Mesh] OR "Pharmacoepidemiology"[tiab] OR "Pharmacovigilance"[tiab] OR "Drug safety"[tiab] OR "Medication safety"[tiab] OR "Adverse drug reaction*"[tiab] OR "Drug surveillance"[tiab]) AND ("Asia"[Mesh] OR "Pacific Islands"[Mesh] OR "China"[Mesh] OR "Japan"[Mesh] OR "Korea"[Mesh] OR "India"[Mesh] OR "Indonesia"[Mesh] OR "Malaysia"[Mesh] OR "Philippines"[Mesh] OR"Singapore"[Mesh] OR "Thailand"[Mesh] OR "Vietnam"[Mesh] OR "Australia"[Mesh] OR "New Zealand"[Mesh] OR "South Pacific"[tiab] OR "Asia Pacific"[tiab] OR "East Asia"[tiab] OR "Northeast Asia"[tiab] OR "Central Asia"[tiab] OR "South Asia"[tiab] OR "Southeast Asia"[tiab] OR "The Pacific"[tiab] OR China[tiab] OR Hong Kong[tiab] OR Macau[tiab] OR "North Korea"[tiab] OR Japan[tiab] OR Mongolia[tiab] OR "South Korea"[tiab] OR Taiwan[tiab] OR Armenia[tiab] OR Azerbaijan[tiab] OR Georgia[tiab] OR Kazakhstan[tiab] OR Kyrgyzstan[tiab] OR Russia[tiab] OR Tajikistan[tiab] OR Turkmenistan[tiab] OR Uzbekistan[tiab] OR Afghanistan[tiab] OR Bangladesh[tiab] OR Bhutan[tiab] OR India[tiab] OR Iran[tiab] OR Maldives[tiab] OR Nepal[tiab] OR Pakistan[tiab] OR "Sri Lanka"[tiab] OR Turkey[tiab] OR Brunei[tiab] OR Cambodia[tiab] OR Indonesia[tiab] OR Laos[tiab] OR Malaysia[tiab] OR Myanmar[tiab] OR Philippines[tiab] OR Singapore[tiab] OR Thailand[tiab] OR "Timor-Leste"[tiab] OR Vietnam[tiab] OR "American Samoa"[tiab] OR Australia[tiab] OR Bougainville[tiab] OR "Cook Islands"[tiab] OR Fiji[tiab] OR "French Polynesia"[tiab] OR Guam[tiab] OR Kiribati[tiab] OR "Marshall Islands"[tiab] OR Micronesia[tiab] OR Nauru[tiab] OR "New Caledonia"[tiab] OR "New Zealand"[tiab] OR Niue[tiab] OR "Northern Mariana Islands"[tiab] OR Palau[tiab] OR "Papua New Guinea"[tiab] OR Samoa[tiab] OR "Solomon Islands"[tiab] OR Tonga[tiab] OR Tuvalu[tiab] OR Vanuatu[tiab])

2. **Scopus**

TITLE-ABS-KEY(("Artificial intelligence" OR "AI" OR "Machine learning" OR "Natural language processing" OR "Deep learning" OR "Neural network*") AND ("Pharmacovigilance" OR "Pharmacoepidemiology" OR "Drug safety" OR "Medication safety" OR "Adverse drug reaction*" OR "Drug surveillance") AND ("South Pacific" OR "Asia Pacific" OR "East Asia" OR "Northeast Asia" OR "Central Asia" OR "South Asia" OR "Southeast Asia" OR "The Pacific" OR "China" OR "Hong Kong" OR "Macau" OR "North Korea" OR "Japan" OR "Mongolia" OR "South Korea" OR "Taiwan" OR "Armenia" OR "Azerbaijan" OR "Georgia" OR "Kazakhstan" OR "Kyrgyzstan" OR "Russia" OR "Tajikistan" OR "Turkmenistan" OR "Uzbekistan" OR "Afghanistan" OR "Bangladesh" OR "Bhutan" OR "India" OR "Iran" OR "Maldives" OR "Nepal" OR "Pakistan" OR "Sri Lanka" OR "Turkey" OR "Brunei" OR "Cambodia" OR "Indonesia" OR "Laos" OR "Malaysia" OR "Myanmar" OR "Philippines" OR "Singapore" OR "Thailand" OR "Timor-Leste" OR "Vietnam" OR "American Samoa" OR "Australia" OR "Bougainville" OR "Cook Islands" OR "Fiji" OR "French Polynesia" OR "Guam" OR "Kiribati" OR "Marshall Islands" OR "Micronesia" OR "Nauru" OR "New Caledonia" OR "New Zealand" OR "Niue" OR "Northern Mariana Islands" OR "Palau" OR "Papua New Guinea" OR "Samoa" OR "Solomon Islands" OR "Tonga" OR "Tuvalu" OR "Vanuatu"))

3. **Google Scholar**

"Artificial intelligence" OR AI OR "Machine learning" OR "Natural language processing" OR "Deep learning" OR "Neural network*" AND "Pharmacovigilance" OR "Pharmacoepidemiology" OR "Drug safety" OR "Medication safety" OR "Adverse drug reaction*" OR "Drug surveillance" AND "South Pacific" OR "Asia Pacific" OR "East Asia" OR "Northeast Asia" OR "Central Asia" OR "South Asia" OR "Southeast Asia" OR "The Pacific" OR China OR "Hong Kong" OR Macau OR "North Korea" OR Japan OR Mongolia OR "South Korea" OR Taiwan OR Armenia OR Azerbaijan OR Georgia OR Kazakhstan OR Kyrgyzstan OR Russia OR Tajikistan OR Turkmenistan OR Uzbekistan OR Afghanistan OR Bangladesh OR Bhutan OR India OR Iran OR Maldives OR Nepal OR Pakistan OR "Sri Lanka" OR Turkey OR Brunei OR Cambodia OR Indonesia OR Laos OR Malaysia OR Myanmar OR Philippines OR Singapore OR Thailand OR "Timor-Leste" OR Vietnam OR "American Samoa" OR Australia OR Bougainville OR "Cook Islands" OR Fiji OR "French Polynesia" OR Guam OR Kiribati OR "Marshall Islands" OR Micronesia OR Nauru OR "New Caledonia" OR "New Zealand" OR Niue OR "Northern Mariana Islands" OR Palau OR "Papua New Guinea" OR Samoa OR "Solomon Islands" OR Tonga OR Tuvalu OR Vanuatu

**Supplementary File 3. Critical Appraisal**

**Table S2. Critical Appraisal of Non-randomised Studies using Modified MINORS**

| **No** | **Study** | **CI Disclosure** | **Study**  **aim** | **Input**  **feature** | **Ground**  **truth** | **Dataset**  **distribution** | **Performance**  **metric** | **AI**  **model** |
| --- | --- | --- | --- | --- | --- | --- | --- | --- |
| 1 | Li et al. | 1 | 1 | 1 | 1 | NA | 1 | 1 |
| 2 | Ye et al. | 0 | 1 | 1 | 1 | NA | 1 | 1 |
| 3 | Yang, H. & Gao, H. | 1 | 1 | 1 | 1 | 1 | 1 | 1 |
| 4 | Chen et al. | 1 | 1 | 1 | 1 | 1 | 1 | 1 |
| 5 | Li et al. | 1 | 1 | 1 | 1 | NA | 1 | 1 |
| 6 | Wang et al. | 0 | 1 | 1 | 1 | 1 | 1 | 1 |
| 7 | Guo et al. | 1 | 1 | 1 | 1 | 1 | 1 | 1 |
| 8 | Wei et al. | 1 | 1 | 1 | 1 | 1 | 1 | 1 |
| 9 | Fu et al. | 1 | 1 | 1 | 1 | 1 | 1 | 1 |
| 10 | Hu et al. | 1 | 1 | 1 | 1 | 1 | 1 | 1 |
| 11 | Feng et al. | 0 | 1 | 1 | 1 | 1 | 1 | 1 |
| 12 | Wei et al. | 1 | 1 | 1 | 1 | 1 | 1 | 1 |
| 13 | Ma et al. | 1 | 1 | 1 | 1 | 1 | 1 | 1 |
| 14 | Zeng et al. | 1 | 1 | 1 | 1 | 1 | 1 | 1 |
| 15 | Kubota et al. | 1 | 1 | 1 | 1 | NA | 1 | 1 |
| 16 | Nomura et al. | 1 | 1 | 1 | NA | NA | 1 | 1 |
| 17 | Noguchi et al. | 1 | 1 | 1 | NA | NA | 1 | 1 |
| 18 | Ujiie et al. | 1 | 1 | 1 | 1 | 1 | 1 | 1 |
| 19 | Ambe et al. | 1 | 1 | 1 | 1 | 1 | 1 | 1 |
| 20 | Matsuda et al. | 1 | 1 | 1 | NA | NA | NA | 1 |
| 21 | Mashima et al. | 1 | 1 | 1 | 1 | 1 | 1 | 1 |
| 22 | Imai et al. | 1 | 1 | 1 | 1 | 1 | 1 | 1 |
| 23 | Fujimoto et al. | 1 | 1 | 1 | 1 | 1 | 1 | 1 |
| 24 | Okamoto et al. | 1 | 1 | 1 | 1 | 1 | 1 | 1 |
| 25 | Maeda-Minami et al. | 1 | 1 | 1 | 1 | NA | 1 | 1 |
| 26 | Watanabe et al. | 1 | 1 | 1 | 1 | 1 | 1 | 1 |
| 27 | Enokiya, T. & Ozaki, K. | 1 | 1 | 1 | 1 | 1 | 1 | 1 |
| 28 | Yada et al. | 1 | 1 | 1 | 1 | NA | 1 | 1 |
| 29 | Kizaki et al. | 1 | 1 | 1 | 1 | 1 | 1 | 1 |
| 30 | Watanabe et al. | 1 | 1 | 1 | 1 | 1 | 1 | 1 |
| 31 | Lou et al. | 1 | 1 | 1 | NA | NA | 1 | 1 |
| 32 | Guan et al. | 1 | 1 | 1 | 1 | NA | 1 | 1 |
| 33 | Kim et al. | 1 | 1 | 1 | 1 | 1 | 1 | 1 |
| 34 | Lee et al. | 1 | 1 | 1 | 1 | 1 | 1 | 1 |
| 35 | Kim et al. | 1 | 1 | 1 | 1 | 1 | 1 | 1 |
| 36 | Jang et al. | 1 | 1 | 1 | 1 | 1 | 1 | 1 |
| 37 | Heo et al. | 1 | 1 | 1 | 1 | 1 | 1 | 1 |
| 38 | Park et al. | 1 | 1 | 1 | 1 | 1 | 1 | 1 |
| 39 | Hu et al. | 0 | 1 | 1 | 1 | 1 | 1 | 1 |
| 40 | Hu et al. | 1 | 1 | 1 | 1 | 1 | 1 | 1 |
| 41 | Wang et al. | 1 | 1 | 1 | 1 | 0 | 1 | 1 |
| 42 | Lai et al. | 1 | 1 | 1 | 1 | 1 | 1 | 1 |
| 43 | Tai et al. | 1 | 1 | 1 | 1 | 1 | 1 | 1 |
| 44 | Ang et al. | 0 | 1 | 1 | 1 | NA | 1 | 1 |
| 45 | Ang et al. | 1 | 1 | 1 | 1 | NA | 1 | 1 |
| 46 | Tang et al. | 1 | 1 | 1 | 1 | 1 | 1 | 1 |
| 47 | Ang et al. | 1 | 1 | 1 | 1 | 1 | 1 | 1 |
| 48 | Jin et al. | 0 | 1 | 1 | 1 | NA | 1 | 1 |
| 49 | Nguyen et al. | 1 | 1 | 1 | 1 | NA | 1 | 1 |
| 50 | Ward et al. | 1 | 1 | 1 | 1 | 1 | 1 | 1 |
| 51 | Wong, E. & Symons, K. | 1 | 1 | 1 | 1 | NA | 1 | 1 |
| 52 | Modayil et al. | 1 | 1 | 1 | 1 | NA | 1 | 1 |
| 53 | Rajalakshmi, P. & Rajagopalan, S.P. | 1 | 1 | 1 | 1 | NA | 1 | 1 |
| 54 | Gurudiwan, P. & Sharma, P. | 0 | 1 | 1 | NA | NA | NA | 1 |
| 55 | Choo et al. | 1 | 1 | 1 | 1 | 1 | 1 | 1 |
| 56 | Basil et al. | 1 | 1 | 1 | 1 | 1 | 1 | 1 |
| 57 | Rekha et al. | 1 | 1 | 1 | 1 | NA | 1 | 1 |
| 58 | Jarynowski et al. | 1 | 1 | 1 | 1 | 1 | 1 | 1 |
| 59 | Hsu et al. | 1 | 1 | 1 | 1 | 1 | 1 | 1 |
| 60 | Yalçın et al. | 1 | 1 | 1 | 1 | 1 | 1 | 1 |
| 61 | Kittisorayut et al. | 0 | 1 | 1 | 1 | 1 | 1 | 1 |
| 62 | Perwitasari et al. | 0 | 1 | 1 | 1 | 1 | 1 | 1 |
| 63 | Vu et al. | 1 | 1 | 1 | 1 | NA | 1 | 1 |

**Table S3. Critical Appraisal of Prediction Modeling Studies using PROBAST**

| **No** | **Study** | **D1 Item1** | **D1 Item2** | **D1 RoB** | **D2 Item1** | **D2 Item2** | **D2 Item3** | **D2 RoB** | **D3 Item1** | **D3 Item2** | **D3 Item3** | **D3 Item4** | **D3 Item5** | **D3 Item6** | **D3 RoB** | **D4 Item1** | **D4 Item2** | **D4 Item3** | **D4 Item4** | **D4 Item5** | **D4 Item6** | **D4 Item7** | **D4 Item8** | **D4 Item9** | **D4 RoB** | **Overall RoB** | **Overall Applicability Concern** |
| --- | --- | --- | --- | --- | --- | --- | --- | --- | --- | --- | --- | --- | --- | --- | --- | --- | --- | --- | --- | --- | --- | --- | --- | --- | --- | --- | --- |
| 1 | Guo et al. | Yes | Yes | Low | Yes | Probably Yes | Yes | Low | Yes | Yes | Yes | Yes | Probably Yes | Yes | Low | Yes | Yes | Yes | No Information | Yes | Yes | Yes | Yes | Yes | Low | Low | Low |
| 2 | Hu et al. | Yes | Yes | Low | Yes | Probably Yes | Yes | Low | Yes | Yes | Yes | Yes | Probably Yes | Yes | Low | Probably Yes | Yes | Yes | No Information | Probably Yes | Yes | Yes | Probably No | Yes | High | High | Low |
| 3 | Zeng et al. | Yes | Yes | Low | Yes | Probably Yes | Yes | Low | Yes | Yes | Yes | Yes | Probably Yes | Yes | Low | Probably No | Yes | Yes | No Information | Probably No | Yes | Yes | Probably No | Yes | High | High | Low |
| 4 | Kim et al. | Yes | Yes | Low | Yes | Probably Yes | Yes | Low | Yes | Yes | Yes | Yes | Probably Yes | Yes | Low | Yes | Yes | Yes | Probably Yes | Yes | Yes | Yes | Probably No | Yes | High | High | Low |
| 5 | Heo et al. | Yes | Yes | Low | Yes | Probably Yes | Yes | Low | Yes | Yes | Yes | Yes | Probably Yes | Yes | Low | Yes | Yes | Yes | Probably Yes | Yes | Yes | Yes | Yes | Yes | Low | Low | Low |
| 6 | Park et al. | Yes | Yes | Low | Yes | Probably Yes | Yes | Low | Yes | Yes | Yes | Yes | Probably Yes | Yes | Low | Probably Yes | Yes | Yes | No Information | Probably Yes | Yes | Yes | Probably No | Yes | High | High | Low |
| 7 | Hu et al. | Yes | Yes | Low | Yes | Probably Yes | Yes | Low | Yes | Yes | Yes | Yes | Probably Yes | Yes | Low | Probably Yes | Yes | Yes | No Information | Probably Yes | Yes | Yes | Probably No | Yes | High | High | Low |
| 8 | Hu et al. | Yes | Yes | Low | Yes | Probably Yes | Yes | Low | Yes | Yes | Yes | Yes | Probably Yes | Yes | Low | Probably Yes | Yes | Yes | No Information | Probably Yes | Yes | Yes | Probably No | Yes | High | High | Low |
| 9 | Tai et al. | Yes | Yes | Low | Yes | Probably Yes | Yes | Low | Yes | Yes | Yes | Yes | Probably Yes | Yes | Low | Probably Yes | Yes | Yes | No Information | Probably Yes | Yes | Yes | Probably No | Yes | High | High | Low |
| 10 | Ward et al. | Yes | Yes | Low | Yes | Probably Yes | Yes | Low | Yes | Yes | Yes | Yes | Probably Yes | Yes | Low | Probably Yes | Yes | Yes | No Information | Probably Yes | Yes | Yes | Probably No | Yes | High | High | Low |
| 11 | Henry et al. | Yes | Yes | Low | Yes | Probably Yes | Yes | Low | Yes | Yes | Yes | Yes | Probably Yes | Yes | Low | Probably Yes | Yes | Yes | Probably Yes | Yes | Yes | Yes | Probably No | Yes | High | High | Low |
| 12 | Hsu et al. | Yes | Yes | Low | Yes | Probably Yes | Yes | Low | Yes | Yes | Yes | Yes | Probably Yes | Yes | Low | Yes | Yes | Yes | Probably Yes | Yes | Yes | Yes | Probably No | Yes | High | High | Low |
| 13 | Yalçın et al. | Yes | Yes | Low | Yes | Probably Yes | Yes | Low | Yes | Yes | Yes | Yes | Probably Yes | Yes | Low | Yes | Yes | Yes | Probably Yes | Yes | Yes | Yes | Probably Yes | Yes | Low | Low | Low |
| 14 | Perwitasari et al. | Yes | Yes | Low | Yes | Probably Yes | Yes | Low | Yes | Yes | Yes | Yes | Probably Yes | Yes | Low | Probably Yes | Yes | Yes | Probably Yes | Probably Yes | Yes | Yes | Probably No | Yes | High | High | Low |

**Table S4. Critical Appraisal for Randomized Study using JBI**

| **No** | **Study** | **True Randomization Used** | **Allocation Concealment** | **Baseline Group Similarity** | **Participant Blinding** | **Treatment Provider Blinding** | **Identical Treatment Aside from Intervention** | **Outcome Assessor Blinding** | **Consistent Outcome Measurement** | **Reliable Outcome Measurement** | **Completeness of Follow-up** | **Intention-to-Treat Analysis** | **Appropriate Statistical Analysis** | **Appropriate Trial Design & Deviation Management** |
| --- | --- | --- | --- | --- | --- | --- | --- | --- | --- | --- | --- | --- | --- | --- |
| 1 | Ye et al. | Yes | Yes | Yes | Yes | Yes | Yes | Yes | Yes | Yes | Yes | Yes | Yes | Yes |

**Supplementary File 4.**

**Table S5. Study Characteristics**

| **No** | **Study** | **Year** | **Settings** | **Focus of Study** | **Data source** | **AI Methods Generic Category** | **Type of Drugs/Diseases** | **Challenges in using AI** | **Recommendations** |
| --- | --- | --- | --- | --- | --- | --- | --- | --- | --- |
| 1 | Li et al | 2008 | China | Signal detection concordance | Guangdong Province Adverse Drug Reaction Database | Statistical Disproportionality Methods | General drugs | AI-based methods like BCPNN require a sufficient number of reports per drug-ADR combination to ensure reliable signal detection | Recommendation for AI use not mentioned |
| 2 | Ye et al | 2009 | China | ADR signal detection | Shanghai Spontaneous Reporting System (SRS) | Statistical Disproportionality Methods | Broad range of drugs | Data preprocessing complexity | Implement automated systems for data acquisition and signal detection |
| 3 | Ye et al | 2018 | China | Drug efficacy evaluation | Department of Neurology of Longhua Hospital and Shuguang Hospital Affiliated to Shanghai University of Traditional Chinese Medicine in China | Neural Networks | Traditional Chinese medicine combined with levodopa/Parkinson’s disease | Challenge in AI use not mentioned, study limitations: limited sample size, lack of generalization to wider population | Recommendation for AI use not mentioned. Recommendation study overall: Combine traditional Chinese and Western medicine approaches for comprehensive PD symptom management. |
| 4 | Yang and Gao | 2018 | China | Medical entity extraction | Text Data from a Prevalent Online Health Community in China | Deep Learning (DL) | General medicines | Manual feature engineering, out-of-vocabulary issues | Implement deep learning models (e.g., CNMER) to enhance medical entity recognition in Chinese online health data. |
| 5 | Chen et al | 2019 | China | ADR extraction and evaluation | Chinese Adverse Drug Event Reports (ADERs) | Machine Learning + Deep Learning (DL) > to enhance NLP tasks | General drugs | Limited annotated data for training | Apply semi-supervised learning techniques to improve NER performance with limited labeled data. |
| 6 | Li et al | 2020 | China | Ontological extraction of ADRs | National Medical Products Administration (NMPA) and other Chinese drug databases | Natural Language Processing (NLP) + Symbolic AI / KRR / Ontology-based | Quinolone antibiotics | Structuring unstructured text data, ensuring accurate biocuration | Expand ontology coverage to include broader drug classes and integrate with intelligent clinical systems. |
| 7 | Wang et al | 2021 | China | Drug safety evaluations | Database of TCM Prescriptions recommended by Chinese authorities for COVID-19 treatment | Neural Networks+Symbolic AI / KRR / Ontology-based | Traditional Chinese Medicine (TCM)/COVID-19 | Risk of overfitting, limited model interpretability | Increase sample size and strengthen AI frameworks to enhance prediction accuracy and reliability. |
| 8 | Guo et al | 2021 | China | Personalized drug concentration prediction | Beijing Anding Hospital | Machine Learning (ML) | Risperidone (antipsychotic) / psychiatric disorders | Limited generalizability from single-center data, lack of pharmacogenetic information | Incorporate diverse datasets and pharmacogenetic variables to improve model accuracy and generalizability. |
| 9 | Wei et al | 2021 | China | Drug risk level prediction | China Food and Drug Administration's Spontaneous Reporting Database (CSRD) | Machine Learning (ML) + Neural Networks | General post-marketing drugs | Class imbalance and classification inaccuracies due to feature representation | Implement GAN and SMOTE-based models to support regulatory decisions and enhance postmarketing surveillance. |
| 10 | Fu et al | 2022 | China | Medication safety through individualized dosing | Clinical data from three transplant centers in China | Machine Learning (ML) | Tacrolimus/kidney transplantation | Incorporating diverse patient factors (genetics, hematocrit, co-medications) into models | Apply the ETR-based model in clinical practice to optimize individualized TAC dosing. |
| 11 | Hu et al | 2023 | China | ADEs prediction model | West China Hospital | Machine Learning (ML) + Neural Networks | Methotrexate / Immune system disorders | Patient data variability, risk of overfitting with limited datasets | Apply AI models clinically to monitor methotrexate and personalize treatment plans. |
| 12 | Feng et al | 2023 | China | Chinese NLP for ADE detection | Real-world clinical data from two tertiary medical institutions in Hunan Provinc | Deep Learning (DL) | General chinese drugs | Complexity of Chinese semantics | Adapt AI models to address missing drug entities and multiple medications in clinical narratives. |
| 13 | Wei et al | 2024 | China | TCM ADR Prediction | Spontaneous reporting data from Jiangsu Province, China | Deep Learning (DL) | Traditional Chinese medicine | Reporting data quality, TCM ingredient complexity, hyperparameter tuning needs | Develop and validate the TIRPnet model with large datasets and integrate it into TCM clinical practice. |
| 14 | Ma et al | 2024 | China | DDI ADR signal detection | Chinese hospital EMRs | Machine Learning (ML) + Natural Language Processing (NLP) | Anti-tuberculosis drugs (Isoniazid) | Data sparsity and computational intensity | Improve data integration strategies to boost AI model performance. |
| 15 | Zeng et al | 2025 | China | ADR risk prediction model | Affiliated Changsha Central Hospital of University of South China | Machine Learning (ML) | Anti-tuberculosis drugs | Potential bias from single-center data, need for external validation | Apply AI models clinically to reduce ATB-DILI and conduct multicenter studies for broader applicability. |
| 16 | Kubota et al | 2004 | Japan | ADR signal detection | Japanese spontaneous ADR database | Statistical Disproportionality Methods | Broad range of drug-ADR combinations | AI-based methods like BCPNN may not detect signals in cases with low report counts, potentially missing rare ADRs | Employ multiple data mining methodologies concurrently to enhance the robustness of ADR signal detection |
| 17 | Nomura et al | 2015 | Japan | Database and signal detection assessment | Japanese Adverse Drug Event Report (JADER) database and the US Food and Drug Administration Adverse Event Reporting System (FAERS), | Statistical Disproportionality Methods | Etanercept, infliximab, and paroxetine hydrochloride | Data variations affecting signal detection | Enhance harmonization of reporting standards and database structures to improve the effectiveness of AI algorithms in pharmacovigilance. |
| 18 | Noguchi et al | 2019 | Japan | Drug-AE relation detection | Spontaneous Reporting System in Japan | Statistical Disproportionality Methods + Other / Unconventional AI Methods | Oral medications/Drug-induced dementia in chronic kidney disease patients | Data quality issues, complex patient profiles, limited model interpretability, need for clinical validation, and high computational demands | Utilize large-scale medical databases for better signal detection |
| 19 | Ujiie et al | 2020 | Japan | Automated ADE article classification | Japanese medical articles, annotated by a medical engineer. | Natural Language Processing (NLP) | General drugs | Need to improve accurate identification of ADE-suggesting sentences | Improve sentence-level classification accuracy by incorporating wider contextual information. |
| 20 | Ambe et al | 2021 | Japan | ADR risk modeling | Japanese Adverse Drug Event Report (JADER) database | Deep Learning (DL) | Drugs associated with Severe Cutaneous Adverse Reactions (SCARs) | Difficulty detecting rare SCARs in trials, model variability outside applicability domain | Incorporate AI models into early drug development and postmarketing safety assessments to identify causative drugs. |
| 21 | Matsuda et al | 2021 | Japan | Patient-centric pharmacovigilance | Japanese health insurance claims database  Tobyoki (Japanese disease blogs) — patient-generated content | Natural Language Processing (NLP) | Systemic Lupus Erythematosus (SLE) related drugs | Unstructured patient narratives lack standardization | Integrate patient-generated data with traditional sources for more comprehensive pharmacovigilance. |
| 22 | Mashima et al | 2022 | Japan | Adverse medication reactions identification | Physicians’ progress notes from Kagawa University Hospital | Natural Language Processing (NLP) | Chemotherapy/Cancer | Ambiguous language, lack of standardized terminologies, complex narratives | Train language models using domain-specific and annotated datasets to improve performance. |
| 23 | Ima i et al | 2022 | Japan | ADR risk factor identification | Large Japanese electronic medical record (EMR) database | Machine Learning (ML) | Vancomycin / Nephrotoxicity | Interpretation and generalization across settings | Utilize AI-derived risk patterns to support targeted pharmacovigilance monitoring. |
| 24 | Fujimoto et al | 2022 | Japan | Early ADR detection | Patient data from Niigata University, Tohoku University, University of Tsukuba, and Osaka University | Deep Learning (DL) | NA (Medical Imaging) | Limited image diversity reduces generalizability, difficulty integrating AI into workflows | Use AI to support early diagnosis, combining results with clinical judgment for improved accuracy. |
| 25 | Okamoto et al | 2023 | Japan | Medical safety evaluation of pharmaceutical products | Japan Council for Quality Health Care (JCQHC) | Machine Learning (ML) | General pharmaceutical products | Human classification accuracy, data quality, model tuning complexity | Improve AI model accuracy and integrate with regulatory agency (PMDA) processes for primary screening. |
| 26 | Maeda-Minami et al | 2023 | Japan | Drug information provision | Package inserts of Kampo medicines  Manuals for Management of Individual Serious Adverse Drug Reactions  Data on off-label usage | Natural Language Processing (NLP) | Kampo medicine | Limited initial data, complex medical terminology, variable user accuracy | Implement AI systems to improve accessibility and accuracy of Kampo medicine information for healthcare professionals. |
| 27 | Watanabe et al | 2024 | Japan | Prediction of clinically significant adverse reaction (CSAR) | Adverse Drug Reaction (ADR) case reports from post-marketing surveillance in Japan | Machine Learning (ML) | Japanese drugs | Inaccurate and incomplete ADR data, challenges in model training and feature incorporation | Refine machine learning models for early safety signal identification and decision support. |
| 28 | Enokiya and Ozaki | 2024 | Japan | ADEs prediction model | JADER database | Machine Learning (ML) | Injection drugs | Limited data scope, exclusion of many drugs due to administration route or analytical issues | Expand data utilization and refine algorithms to improve model prediction and applicability. |
| 29 | Yada et al | 2024 | Japan | Text mining for medical RWD | Electronic health records (EHRs)  Medication orders  Discharge summaries (unstructured text) from a Japanese Hospital | Natural Language Processing (NLP) | Japanese cancer-related drugs | Difficulty extracting medical information from unstructured text, need for high-quality data | Utilize textual information extraction and develop practical systems to support real-world data mining. |
| 30 | Kizaki et al | 2024 | Japan | Medication safety evaluation | Incident reports from residential care facilities in Japan | Natural Language Processing (NLP) | General medications | Complex incident reports, need for annotated data, low inter-annotator agreement | Refine NLP models and integrate them into incident reporting systems to improve medication safety. |
| 31 | Watanabe et al | 2024 | Japan | ADR label changes prediction | Japanese Adverse Drug Event Report (JADER)  U.S. FDA Adverse Event Reporting System (FAERS) | Machine Learning (ML) | General drugs | Data variability (domestic vs. overseas ADR reports), model accuracy limitations | Improve model robustness by leveraging diverse datasets. |
| 32 | Lou et al | 2025 | Japan | ADE signal detection | Japanese Adverse Drug Event Report (JADER)  U.S. FDA Adverse Event Reporting System (FAERS) | Statistical Disproportionality Methods | Paliperidone palmitate/Schizophrenia and schizoaffective disorder | Not discussed | Use of multiple pharmacovigilance databases (FAERS and JADER) enhances the robustness of signal detection.  Combining multiple statistical methods increases confidence in ADE signals.  Further clinical evaluation of unexpected signals is needed. |
| 33 | Guan et al | 2023 | China, Japan | ADR signal detection | Jinan Adverse Event Reporting System (JAERS), China | Statistical Disproportionality Methods | Cardiac therapy drugs / Cardiovascular conditions | Data quality, underreporting, and lack of causality verification | Combine multiple algorithms and strengthen pharmacovigilance practices to improve ADR signal detection |
| 34 | Kim et al | 2021 | South Korea | Vaccine-related adverse events surveillance | Korea Disease Control and Prevention Agency (vaccination records)  National Health Insurance Service (NHIS), Korea | Machine Learning (ML) + Explainable AI (XAI) | Influenza vaccine | Limited model generalizability due to small sample and demographic scope | Integrate machine learning models into national pharmacovigilance systems for earlier detection of vaccine-related ADRs. |
| 35 | Lee et al | 2022 | South Korea | ADR signal detection | Korea Adverse Event Reporting System (KAERS)  Also used FAERS for validation/application | Machine Learning (ML) | Infliximab/rheumatoid arthritis | Data sparsity, model generalization across datasets | Improve training data and enhance algorithm robustness. |
| 36 | Kim et al | 2023 | South Korea | Drug safety data extraction and enhancement | Individual Case Safety Reports (ICSRs) reported through KAERS (Korea Adverse Event Reporting System) | Natural Language Processing (NLP) | General drugs | Limited generalizability, Korean language complexity, narrative variability | Improve model robustness with diverse datasets and integrate NLP systems in pharmacovigilance pipelines. |
| 37 | Jang et al | 2023 | South Korea | ADR signal detection | KAERS (Korea) | Machine Learning (ML) | Fluoroquinolones | Data quality, model tuning complexity, integration with pharmacovigilance systems | Implement tree-based machine learning methods for better pharmacovigilance signal detection. |
| 38 | Heo et al | 2024 | South Korea | ADR prediction model | Electronic Health Records (EHRs) from six hospitals in Korea | Deep Learning (DL) | Angiotensin II receptor blockers (ARBs) and drug-induced liver injury (DILI). | Class imbalance, generalizability, data consistency, black-box AI models | Apply AI models in clinical settings to enable early detection of DILI in patients prescribed ARBs. |
| 39 | Park et al | 2025 | South Korea | ADR prediction model | Electronic medical records in the Observational Medical Outcomes Partnership–Common Data Model (OMOP-CDM) format from two South Korean hospitals (KHMC and KHNMC). | Machine Learning (ML) | Cancer immunotherapy drugs (PD-1/PD-L1 inhibitors) | Data harmonization, model generalizability | Expand data sources and validate models across multiple institutions. |
| 40 | Hu et al | 2012 | Taiwan | Dosage prediction model | Historical clinical records of patients in Taiwan. | Machine Learning (ML) | Warfarin | Limited generalizability due to single-center data, exclusion of genetic factors | Integrate diverse datasets and genetic data to improve AI model accuracy and applicability. |
| 41 | Hu et al | 2018 | Taiwan | Dosage prediction model | Medical center in Taiwan | Machine Learning (ML) | Digoxin/Congestive Heart Failure (CHF) | Limited generalizability and clinical flexibility due to single-institution dataset and fixed digoxin dose focus | Use broader datasets and variable dose modeling to improve model applicability and accuracy. |
| 42 | Wang et al | 2019 | Taiwan | ADR prediction model | Side Effect Resource (SIDER) | Deep Learning (DL) | General drugs | Dependence on input data quality; challenges capturing rare or complex ADRs | Integrate deep learning with rich biomedical data to enhance early ADR detection. |
| 43 | Lai et al | 2020 | Taiwan | ADR prediction model | Clinical and genomic data from Taipei Medical University-Wanfang Hospital (training) and Taipei Medical University-Shuang Ho Hospital (test), Taiwan. | Machine Learning (ML) + Neural Networks | Anti-tuberculosis medications | Small sample size, single-center data limit generalizability | Conduct larger, multicenter studies to support broader clinical validation. |
| 44 | Tai et al | 2020 | Taiwan | Risk prediction of high-alert medication | Medical records of 513 hospitalized patients treated with digoxin at a tertiary medical center in Taiwan. | Machine Learning (ML) | Digoxin/cardiovascular diseases | Limited generalizability from single-center data, focus on major drug interactions, interpretability limitations | Validate model generalizability, expand DDI coverage, and enhance interpretability to build clinician trust. |
| 45 | Ang et al | 2016 | Singapore | ADE corpus annotation framework | Discharge summaries from a tertiary hospital in Singapore. | Natural Language Processing (NLP) | General drugs | Low inter-annotator agreement on drug-adverse event relationships | Use machine pre-annotation tools to streamline drug-related annotation processes. |
| 46 | Ang et al | 2016 | Singapore | AE signal identification | Spontaneous adverse event (AE) reports submitted to Health Sciences Authority, Singapore, | Statistical Disproportionality Methods | Broad range of drug-ADR combinations | BCPNN may have lower sensitivity compared to other methods, potentially missing some safety signals. | Combine AI-based and traditional methods for more robust safety signal detection |
| 47 | Tang et al | 2019 | Singapore | Detection of Drug-AE relations | Hospital discharge summaries from a tertiary hospital in Singapore | Natural Language Processing (NLP) | General drugs | Performance variability from complex clinical narratives | Regularly update drug and AE dictionaries and iteratively refine rule-based systems. |
| 48 | Ang et al | 2021 | Singapore | Detection of substandard medicine alerts | Web alerts on substandard medicines automatically crawled from 46 web pages of 9 regulatory agencies | Machine Learning (ML) | NA (Product Safety) | Ensuring data quality and relevance from diverse online sources | Use AI-driven web surveillance in regulatory settings to proactively identify product-related risks. |
| 49 | Jin et al | 2008 | Australia | Unexpected temporal pattern mining | Real-world healthcare administrative databases | Other / Unconventional AI Methods | Broad spectrum of drugs | Traditional data mining techniques struggle with identifying unexpected and infrequent ADRs due to their design for frequent pattern detection | Implement algorithms like MUTARC that are tailored to uncover unexpected temporal associations in healthcare data for improved ADR detection |
| 50 | Nguyen et al | 2017 | Australia | Social media ADR estimation | Social media platforms (Twitter, Reddit, LiveJournal); side effect data from SIDER database. | Neural Networks | Psychiatric medications | Variability in social media language and context complicates ADR detection | Leverage social media monitoring in pharmacovigilance systems to detect ADRs early. |
| 51 | Ward et al | 2021 | Australia | Adverse outcomes prediction model | Linked Western Australian administrative health datasets | Explainable AI (XAI) | Musculo-skeletal system drugs (ATC class M) and Cardiovascular system drugs (ATC class C) | Data integration complexity | Further develop explainable AI (XAI) algorithms to enhance pharmacovigilance monitoring. |
| 52 | Wong and Symons | 2024 | Australia | Product information translation | Foreign language Product Information (PI) documents for five imported medicines | Natural Language Processing (NLP) | General injectable medicines | Translation inaccuracies, need for human verification | Apply machine translation with rigorous validation and cross-referencing to ensure linguistic accuracy. |
| 53 | Modayil et al | 2010 | India | ADR and risk factors identification | Spontaneous ADR reports by clinicians | Statistical Disproportionality Methods | Antiretroviral therapy for HIV/AIDS. | Complexity of accurately detecting ADR signals | Implement continuous monitoring and reporting systems for ADRs to enhance patient safety and optimize antiretroviral therapy outcomes. |
| 54 | Rajalakshmi and Rajagopalan | 2019 | India | Monitoring of ADRs | Online drug reviews and websites | Natural Language Processing (NLP) | General drugs | Ambiguous natural language, inconsistent user data, difficulty in sentiment assignment | Advance semantic processing and integrate structured and unstructured data for better ADE detection. |
| 55 | Gurudiwan and Sharma | 2024 | India | ADR data extraction & management | FAERS (FDA Adverse Event Reporting System) dataset | Natural Language Processing (NLP) | Acetaminophen and ibuprofen | ADR underreporting | Promote ADR reporting among healthcare professionals and the general public. |
| 56 | Choo et al | 2024 | Malaysia | AE report completeness factors | VigiBase (WHO global database of reported potential adverse events) | Machine Learning (ML) | General drugs reported in Malaysia | Inconsistent data quality, lack of standardized quality definitions, need for interpretable models | Leverage AI to enhance report quality, prioritize interpretable models, and standardize global reporting. |
| 57 | Henry et al | 2024 | Malaysia | Risk prediction of medication administration errors (MAEs) | Direct observational data from five public hospitals in Malaysia (NICUs) | Machine Learning (ML) | Medications used in neonatal intensive care | Data quality, workflow integration, model generalizability | Conduct multicenter studies to validate models and ensure real-world integration and sustainability. |
| 58 | Rekha et al | 2024 | Malaysia | ADR signal detection | Electronic ADR database from Malaysia’s:  National Pharmaceutical Regulatory Agency (NPRA) | Statistical Disproportionality Methods | Pediatric ADRs (e.g., paracetamol, ibuprofen, amoxicillin, metoclopramide). | Underreporting, selective reporting, and lack of digital infrastructure | Enhance digital infrastructure, adopt surveillance tools, prioritize pediatric safety signals. |
| 59 | Jarynowski et al | 2021 | Russia | Vaccine-related AEs identification | Self-reported adverse events (AEs) from users in a Telegram group (Russian language) | Natural Language Processing (NLP) | Sputnik V COVID-19 vaccine | Potential bias from self-reported data and social media demographics | Incorporate social media analytics into pharmacovigilance for earlier detection of vaccine-related adverse events. |
| 60 | Hsu, W et al | 2022 | New Zealand | Prediction of medication adherence | Patient data from a New Zealand CVD risk assessment tool linked routinely collected national and regional datasets | Neural Networks | Lipid-lowering therapy / Cardiovascular disease | Need for large datasets, temporal model complexity, integration of patient history | Use deep temporal models to improve prediction of medication adherence. |
| 61 | Yalçın et al | 2023 | Turkey | Medication error prediction model | Medication orders and patient records from a NICU (Neonatal Intensive Care Unit) in Ankara, Turkey | Machine Learning (ML) | Medications used in neonatal intensive care | Data quality, model generalizability, integration into existing workflows | Validate AI systems in diverse hospital settings and integrate them into NICU workflows. |
| 62 | Kittisorayut et al | 2024 | Thailand | Drug identification model | A manually collected dataset of commonly used drugs in Thailand | Other / Unconventional AI Methods | General drugs | Data quality, algorithm accuracy, integration with healthcare systems | Advance AI algorithm development and integrate systems with healthcare infrastructure to enhance medication safety. |
| 63 | Perwitasari et al | 2025 | Indonesia | Prediction of medication adherence | Prospective longitudinal data from adult tuberculosis patients in Indonesia | Machine Learning (ML) + Neural Networks | First-line anti-tuberculosis drugs | Small sample size due to COVID-19, exclusion of baseline and comorbidity data | Incorporate education on hepatotoxicity, behavioral factors, and broadly validate predictive models. |
| 64 | Vu et al | 2025 | Vietnam | Drug classification | Vietnamese drug dataset | Machine Learning (ML) + Deep Learning (DL)/Neural Nerworks | General drugs | Sparse and imbalanced data | Improve data preprocessing and fine-tune AI models. |
